# sRNAs as possible regulators of retrotransposon activity in *Cryptococcus gattii* VGII

**DOI:** 10.1186/s12864-017-3688-4

**Published:** 2017-04-12

**Authors:** Patrícia Aline Gröhs Ferrareze, Rodrigo Silva Araujo Streit, Francine Melise dos Santos, Augusto Schrank, Livia Kmetzsch, Marilene Henning Vainstein, Charley Christian Staats

**Affiliations:** 1 Programa de Pós-Graduação em Biologia Celular e Molecular, Centro de Biotecnologia, Universidade Federal do Rio Grande do Sul (UFRGS), 91501-970 Porto Alegre, RS Brazil; 2 Departamento de Biologia Molecular e Biotecnologia, Instituto de Biociências, Universidade Federal do Rio Grande do Sul (UFRGS), Porto Alegre, RS Brazil

**Keywords:** *Cryptococcus gattii*, RNAi, Retrotransposons, Synteny

## Abstract

**Background:**

The absence of Argonaute genes in the fungal pathogen *Cryptococcus gattii* R265 and other VGII strains indicates that yeasts of this genotype cannot have a functional RNAi pathway, an evolutionarily conserved gene silencing mechanism performed by small RNAs. The success of the R265 strain as a pathogen that caused the Pacific Northwest and Vancouver Island outbreaks may imply that RNAi machinery loss could be beneficial under certain circumstances during evolution. As a result, a hypermutant phenotype would be created with high rates of genome retrotransposition, for instance. This study therefore aimed to evaluate *in silicio* the effect of retrotransposons and their control mechanisms by small RNAs on genomic stability and synteny loss of *C. gattii* R265 through retrotransposons sequence comparison and orthology analysis with other 16 *C. gattii* genomic sequences available.

**Results:**

Retrotransposon mining identified a higher sequence count to VGI genotype compared to VGII, VGIII, and VGIV. However, despite the lower retrotransposon number, VGII exhibited increased synteny loss and genome rearrangement events. RNA-Seq analysis indicated highly expressed retrotransposons as well as sRNA production.

**Conclusions:**

Genome rearrangement and synteny loss may suggest a greater retrotransposon mobilization caused by RNAi pathway absence, but the effective presence of sRNAs that matches retrotransposon sequences means that an alternative retrotransposon silencing mechanism could be active in genomic integrity maintenance of *C. gattii* VGII strains.

**Electronic supplementary material:**

The online version of this article (doi:10.1186/s12864-017-3688-4) contains supplementary material, which is available to authorized users.

## Background

Transposable elements (TE) are repeated DNA sequences that move and propagate in genome by “copy-and-paste” (TE class I) and “cut-and-paste” (TE class II) mechanisms [1]. TE class I, or retrotransposons, replicate through an RNA intermediate. Autonomous sequences encode their own reverse transcriptase and are typically 5–10 kilobases in length. There are two classes of retrotransposons, based on the presence of LTR (long terminal repeats) or its absence (non-LTR retrotransposons – LINE/SINE elements) [2]. At each end of LTR retrotransposons, there are repeats necessary for their replication cycle which contain sequences that regulate transcription. All LTR retrotransposons can have *gag* and *pol* genes; *gag* encodes proteins (GAGs; Group-specific AntiGen) that form VLPs (Virus-Like Particles), and *pol* encodes a polypeptide composed of (i) integrase, (ii) ribonuclease H, (iii) reverse transcriptase, and (iv) aspartyl protease enzymes [3]. *Gag* and *pol* genes are usually located in two open reading frames (ORFs), but in some LTR retrotransposons, a single *gag*/*pol* ORF is separated by a frameshift or a stop codon [4].

Transposable elements most commonly found in fungal species are retrotransposons. Some studies have elucidated significant effects of transposable elements as an evolutionary force, leading to synteny loss, avirulent gene deletion, and genome expansion [1, 5]. Insertion of transposable elements near genes can lead to changes in gene expression patterns, while insertions within them can modify gene structure. Recombination between LTR elements in different sites leads to chromosomal rearrangements on a large scale. Because of their potentially deleterious effects, host organisms frequently evolve mechanisms to limit the activity of such elements [2].

The function of small RNAs as a defense mechanism against mobile elements is widely conserved among double-stranded RNA-mediated interference (RNAi) routes [6]. In recent years, several studies have examined the role of miRNAs in various cellular processes such as genomic integrity maintenance, DNA damage responses to biotic stress, and morphological process regulation. Interfering RNAs play critical roles in gene regulation, chromosomal structure, and genomic stability, such that the RNAi conserved function is to protect the genome from mobile element invasion [6]. The RNAi pathway mediates homology-dependent degradation of an mRNA with small RNA molecules and is a key regulatory mechanism that controls transcription and translation in eukaryotic organisms. The enzymes required for RNA silencing are numerous and may vary among species, but universally include Argonaute proteins that bind small RNAs, Dicer ribonuclease that produces small interfering RNAs (siRNA) from double stranded RNA precursors (dsRNA), and RNA-dependent RNA polymerase that produces dsRNAs [7]. In this process, a double stranded RNA produced by an RNA-dependent RNA polymerase is first processed into small interfering RNAs of 21–28 nucleotides by Dicer RNase III and enters the canonical pathway. This siRNA is incorporated into a nuclease complex, the RNA-induced silencing complex (RISC), containing an Argonaut protein with RNA-degrading activity. After the RISC complex, siRNAs promote the cleavage of homologous mRNAs [8]. These factors play a key role in eukaryote genome defense: null mutations in their corresponding genes result in increased transposons expression and mobilization, decrease and loss of endogenous retrotransposon-derived siRNAs, and drug resistance mutations induced by retrotransposons [9–11].

Two species from the genus *Cryptococcus* are the etiologic agent of cryptococcosis. *Cryptococcus neoformans* has worldwide distribution and affects immunocompromised individuals, mainly causing cryptococcal meningoencephalitis. *Cryptococcus gattii*, prior to the Pacific Northwest outbreak, was known to be endemic in tropical and subtropical areas, typically infecting healthy hosts with pulmonary cryptococcosis [12, 13]. Estimated numbers calculate that *C. neoformans* cryptococcosis kills 650,000 immunocompromised HIV/AIDS patients every year worldwide [13]. However, the common clinical indistinction between *C. neoformans* and *C. gattii* during cryptococcosis diagnosis can underestimate the real number of cases associated with *C. gattii* cryptococcosis. Studies in a murine model of pulmonary cryptococcosis have demonstrated that there is no significant difference in mortality rates of mice infected with *C. neoformans* H99 and the hypervirulent strain *C. gattii* R265 [14].

The *C. neoformans* serotype D contains two paralogous genes for Argonaute (*AGO1* and *AGO2*) and Dicer (*DCR1* and *DCR2*), and one gene for RdRP (*RDP1*). In contrast, serotype A has a single Argonaut gene (*AGO1*) and RdRP (*RDP1*) and two paralogous genes for Dicer (*DCR1* and *DCR2*). *C. gattii* has four molecular types (VG “variety gattii”): VGI and VGII are the main pathogens of cryptococcosis in immunocompetent hosts, while VGIII and VGIV rarely cause infections and affect immunocompromised hosts [15]. VGI, VGIII, and VGIV genotypes have all the components of an RNAi system, while VGII has a canonical *DCR2* and *DCR1/RDP1* pseudogenes [16]. Bielska and May [13] have shown that 97% of documented cases of cryptococcosis in the Vancouver Island outbreak were caused by VGII, with VGIIa prevalent in more than 80% of the cases.

The absence of Argonaute genes in the primary pathogen *C. gattii* R265, and other VGII strains, indicates that these genotypes do not have a functional RNAi pathway [16] . The success of the R265 strain as a pathogen, in causing the Vancouver Island and Pacific Northwest outbreaks, may imply that RNAi machinery loss can be beneficial under certain circumstances during evolution [6]. This can be linked to the higher recombination rates of VGII strains, which are associated with the generation of novel and possibly more virulent genotypes [17]. The loss of RNAi could thus allow greater mobility of transposable elements, as has been reported in *C. neoformans* [18]. However, in the RNAi-lacking budding yeast *Saccharomyces cerevisiae*, a predominant proportion of sequenced small RNAs mapped to transposable elements [5]. Recent analysis showed that some yeasts, which have presumably lost the RNAi route, actually have siRNAs and use non-canonical Dicer proteins to control Argonaute-like proteins without RdRP [8]. Therefore, an alternative retrotransposon silencing mechanism by sRNAs may act through a non-canonical RNAi pathway or through independent control systems.

Thus, the study of retrotransposon effects in *C. gattii* genomes, as a hypervirulent strain, with potentially high activity of transposable elements unrelated to any silencing mechanism, may contribute to increased understanding of the role of retrotransposons and sRNAs in genome evolution.

## Results

### Retrotransposon mining in *C. gattii* genome sequences

**Fig. 1:**
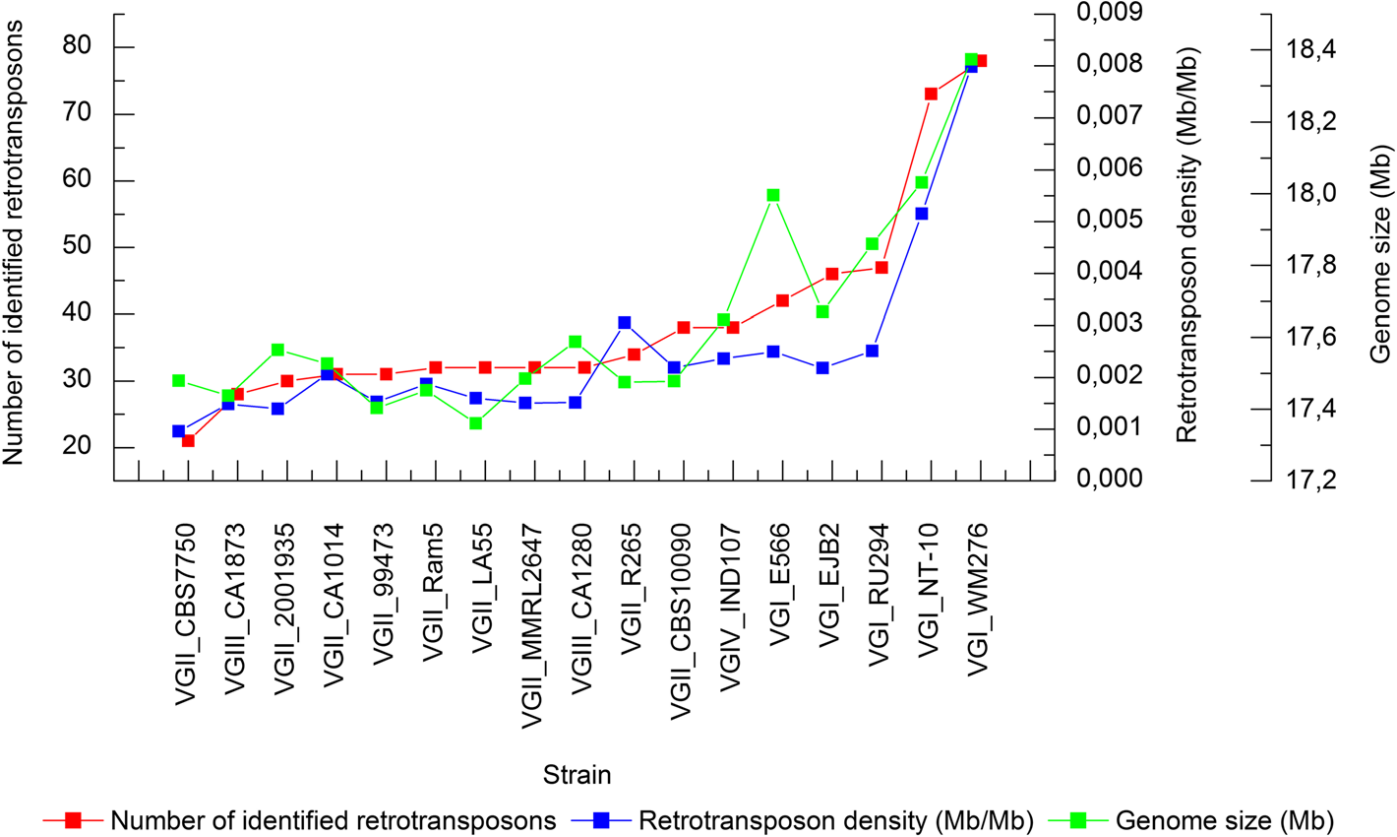
Retrotransposon number, density and genome size comparison. The *red line* indicates the number of identified retrotransposons in each strain; *blue line* represents retrotransposon density in *C. gattii* genomes. The *green line* shows the size of *C. gattii* assembled genomes

Retrotransposon structure usually consists of two almost identical LTRs flanking two ORFs to the *gag* and *pol* genes [19]. Retrotransposon mining programs rely on the search for several structural features of LTR retrotransposons, such as the size range of the LTR sequences, the distance between the two LTRs of an element, among others [20]. The number of LTR-retrotransposons identified in 17 *C. gattii* genomes ranged from 21 to 78 sequences (Fig. 1). Their structural composition was confirmed using NCBI Blast (Conserved Domains) for the presence of *gag* and *pol* domains (Additional file 1). In this search, non-autonomous LTR sequences (which do not encode necessary proteins for transposition) were excluded. Reciprocal BLASTn (data not shown) and domain arquitecture present in the predicted proteins (Additional file 1) revealed that such sequences groups are unique. As shown in Fig. 1, VGI strains (WM276, NT10, E566, EJB2, and RU294), which putatively contain a complete RNAi pathway, present a higher retrotransposon count (especially WM276 and NT10) when compared to VGII (R265, CA1014, CBS7750, CBS10090, RAM5, LA55, MMRL2647, 99473, and 2001935), VGIII (CA1280 and CA1873), and VGIV (IND107) strains.

### Retrotransposon orthology and synteny evaluation

Retrotransposon integration is a non-random process in the genome, as many transposable elements integrate into specific sequences as intergenic regions or upstream of RNA polymerase III-transcribed genes [21]. *Copia* elements (Ty1 family: Pseudoviridae) present specificity for pre-existing LTR transposons (resulting in a TE cluster formation) and transcriptionally active regions, while *gypsy* elements (Ty3 family: Metaviridae) are reported in heterochromatin [22]. Preferential insertion of TE clusters in regions with low recombination rates can be explained by weak selection against these sites [21]. For these reasons, most filamentous fungi carry more Ty3/*gypsy* than Ty1/*copia* elements [22]. The presence of retrotransposon-enriched regions (TE clusters) can be observed by sequence distribution along the 14 chromosomes of WM276 (Additional file 1). However, the 73 retrotransposon elements detected in the NT10 genome are generally incomplete (absence of conserved domain) and dispersed along the genome. A slight prevalence of Ty3/*gypsy* was found in the element composition of *C. gattii* strains.

**Fig. 2:**
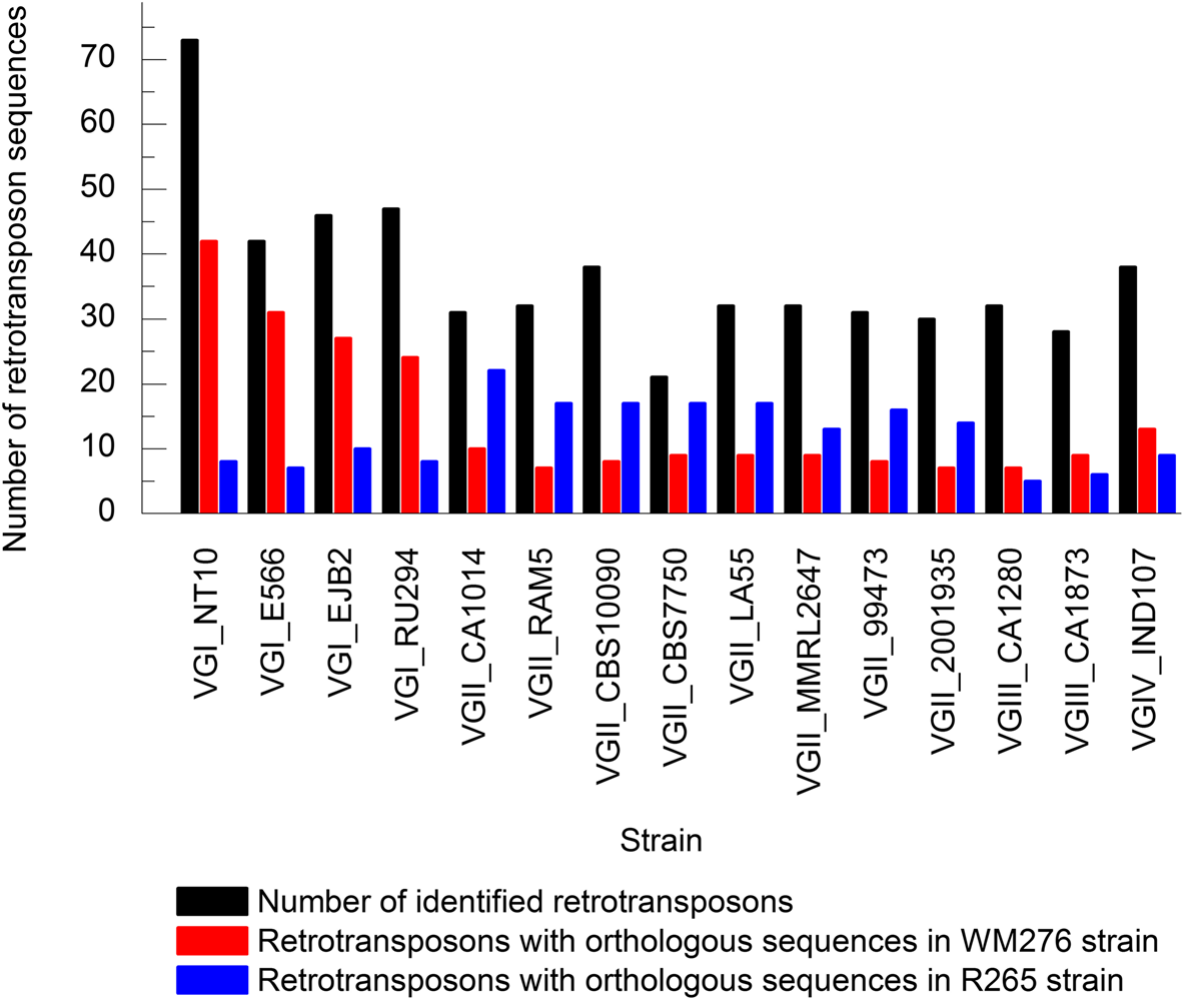
Orthologous sequences count. Orthologous sequences from *C. gattii* WM276 and R265 shared with VGI, VGII, VGIII, and VGIV strains

The WM276 strain has 78 retrotransposon sequences, of which 24 are unique and 29 shared only with VGI strains. The R265 strain has 34 sequences, five of which are unique with 14 exclusive to VGII strains. Unique sequences indicate recent transposition events, since they do not have identifiable orthologues. Figure 2 shows the relationship between sequence amount and orthologue number. VGII strains (mainly CA1014) share more sequences with R265 than other genotypes. Similarly, WM276 shares 49 sequences with VGI strains. These results indicate the existence of transposition events after genotype diversification. Data analysis has allowed the observation of the prevalence (almost exclusivity) of *gag* domains in Ty1 family sequences in the absence of aspartic protease. However, Ty3 has aspartic protease domains and is lacking *gag*. The presence of RVT_1 (Pfam: PF00078) and RVT_2 (Pfam: PF07727) reverse transcriptase families have been observed in *gypsy* and *copia*, respectively.

The chromodomains identified next to retrotransposons WM276.1, 53, 72, and 78 can be related to the *Chromovirus* genus of the Metaviridae (Ty3) family. These chromodomains, absent in R265 strain, are found in functional proteins as well as *gypsy* LTR retrotransposons. The canonical function includes chromatin remodeling and gene expression regulation. However, chromodomains in retrotransposons are poorly understood [23].

Genome rearrangements naturally accumulate in different strains. Successive rearrangements in *C. gattii* and *C. neoformans* genomes may have contributed to sexual isolation and speciation [24]. To determine the effect of retrotransposon mobilization in VGI and VGII strains, their synteny conservation was evaluated (Additional file 1). Orthologous elements shared by VGI and VGII genotypes were analyzed for synteny on a multiple genome alignment. Multiple alignments were performed using the Mauve program with WM276 as a reference for supercontig reordering. VGI strain alignment identified 29 synteny blocks in WM276 (Additional file 2) while VGII alignment found 54 synteny blocks in R265 (Additional file 3), indicating greater genome fragmentation or a better VGI genome sequence assembly. Both alignments had few rearrangements, with a predominant collinear genome. Coordinates of orthologous retrotransposons were used to classify syntenic and non-syntenic sequences.

Long terminal repeats from LTR retrotransposons are known to promote crossing over at non-homologous sites, leading to chromosomal rearrangement. Likewise, mutations arising from mobile element insertions in conserved blocks can cause synteny loss [21]. TE clusters from WM276 were shown to be responsible for synteny breaks at 5 genome points (Additional files 4, 5, 6, 7 and 8). The same number was found in R265 (Additional files 9, 10, 11, 12 and 13), suggesting that R265 active retrotransposons, even if in a small identifiable number, are more influential on genome integrity than WM276 sequences.

**Fig. 3:**
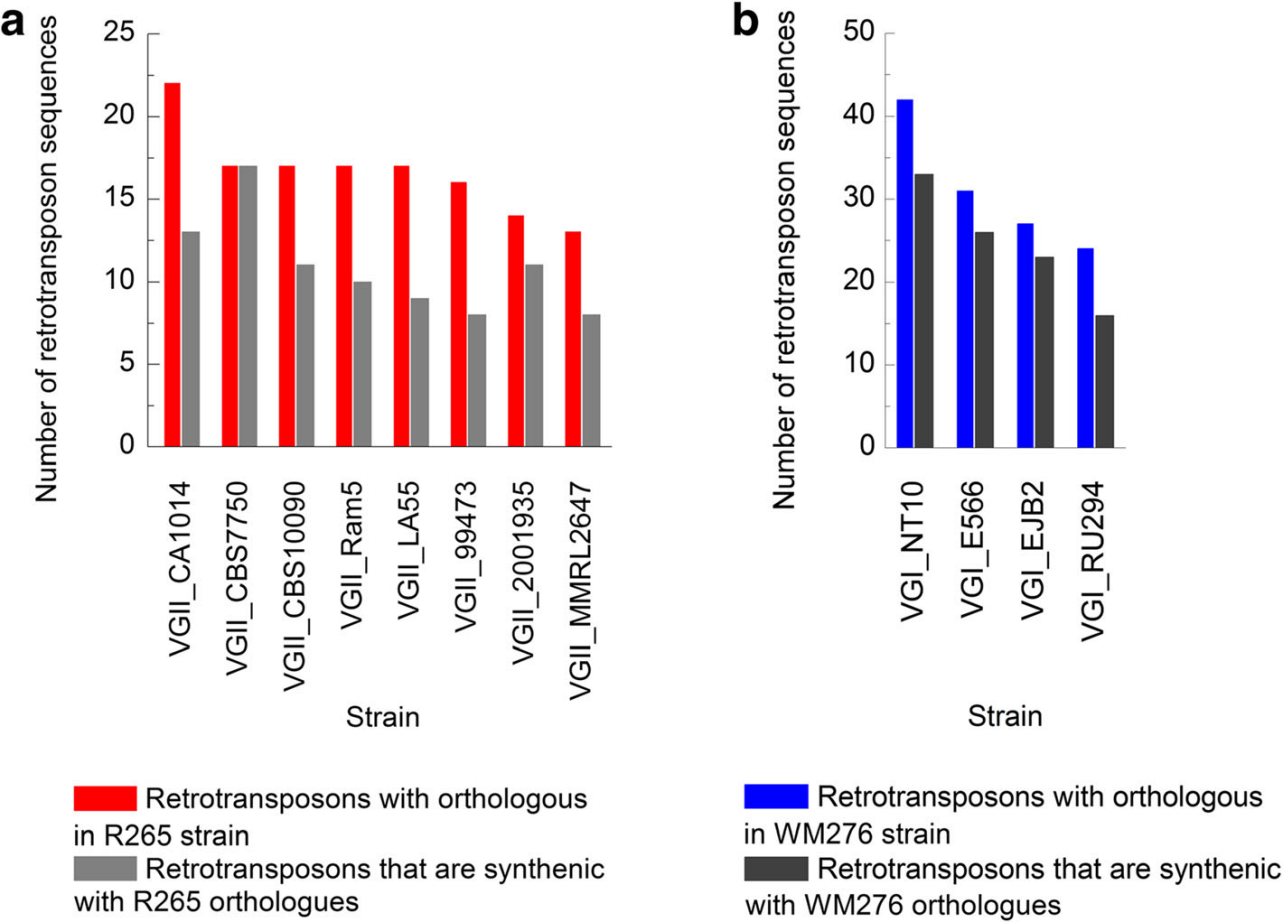
Sequence count of orthologous and syntenic retrotransposons. *Bars* indicate the total amount of orthologous and syntenic sequences shared by VGII (**a**) and VGI (**b**) strains with R265 and WM276 genomes, respectively

*C. gattii* R265 strain retrotransposons have 133 orthologous sequences in 8 VGII genomes. The majority of these (65.41%) preserve synteny with the R265 genome (Fig. 3a), particularly the CBS7750 strain, which has 17 orthologous sequences in syntenic blocks with R265. In comparison, VGI, despite its higher elements count, has almost 80% of orthologous sequences in synteny with the WM276 genome (Fig. 3b).

Among the widely distributed orthologous sequences of R265.3, R265.19, and R265.22 retrotransposons, the multiple genome alignment of 17 *C. gattii* strains shows synteny conservation for most of the *C. gattii* strains (Additional file 14). However, some variations suggest genome rearrangement events (Additional file 15). Orthologs of retrotransposon R265.3 identified in RU294 (VGI), IND107 (VGIV), and CA1280 (VGIII) are localized in the same block and generate their own syntenic group, while orthologs of retrotransposon R265.19 are kept syntenic in all genomes of these three genotypes. Among VGII, orthologs of retrotransposon R265.3 and orthologs of retrotransposon R265.19 sequences were located in opposite positions, such as an ectopic recombination result. This sequence inversion was found in CA1014, CBS10090, MMRL2647, and 2001935. Moreover, CA1014 and CBS10090 each present an exclusive syntenic location to sequence orthologs of retrotransposon R265.22.

Synteny conservation at retrotransposon orthologous sites indicates low mobilization rates and reveals that an RNAi pathway effectively acts in WM276 to protect the genome against retrotransposon propagation. In this context, it is feasible to assume that there are additional mechanisms protecting the VGII strain genomes from mobile elements.

### Phylogenetic inference

**Fig. 4:**
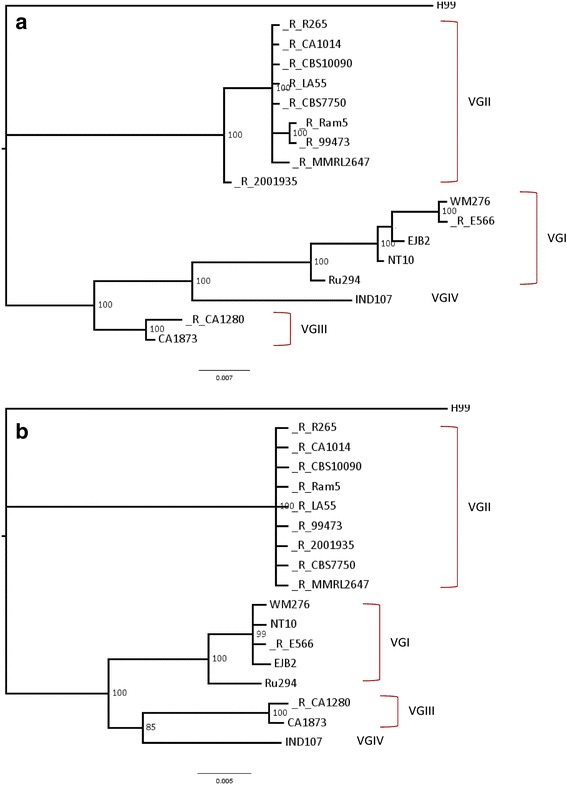
Phylogenetic tree for reverse transcriptase (RT) and RNase H domains. Domains of reverse transcriptase (**a**) and ribonuclease H (**b**) from *C. gattii* retrotransposons to orthologous R265.19 sequence. Sequences that were direction-adjusted by MAFFT aligner are indicated by the “R” before strain name. Model inference was performed by jModelTest and phylogeny reconstruction by Mr. Bayes using the Kimura-2-parameters substitution model

Among the 34 retrotransposons belonging to the R265 genome, three RT sequences (named R265.3, R265.19, and R265.22) have orthologues present in all 17 *C. gattii* strains. Of these, only one (R265.19) was retained in *C. neoformans* H99. The RT R265.19 has two conserved domains (reverse transcriptase and RNase H) in all orthologous sequences (except in H99, where the sequence is inserted in a TE cluster) and was used as a model for the phylogenetic evaluation of *C. gattii* retrotransposon propagation. The evolutionary model selected for nucleotide substitution was Kimura 2-parameters [25]. The phylogram (Fig. 4) shows *C. gattii* diversification through the evolutionary path taken by reverse transcriptase (Fig. 4a) and RNase H (Fig. 4b) sequences. In both cases, monophyletic clades for 4 *C. gattii* genotypes are identifiable. Although the reverse transcriptase domain seems to have undergone more modifications, both trees indicate the relationship between VGI, VGIII, and VGIV, as well as the basal separation of the VGII clade. Early divergence and isolation of VGII molecular types as well as the late separation of VGI, VGIII, and VGIV from a common ancestor has been proposed by Farrer and colleagues [15] in an evolutionary relationship reconstruction for *C. gattii* genotypes.

### Expression evaluation

The activation of transposable elements in response to stress conditions usually involves regulatory sequences at promoter regions, which are similar to motifs required for the induction of stress-responsive genes [26] as well as the preferential integration of some type of retrotransposon sequence into the promoters of stress response genes [27]. Different stress conditions, such as ionizing radiation, reactive oxygen species (ROS), DNA damage, and nutrient starvation can activate transcription and retrotransposition [26, 28]. Hence, element mobilization provide a means for the rapid evolution of gene regulatory networks, mediating the adaptive response to stress conditions [27]. Therefore, we speculated that stress conditions caused by zinc starvation in wild-type (WT) cells and cells lacking the *ZAP1* gene mutant would alter retrotransposon expression. Zinc restriction was show to induces oxidative stress in yeast cells [29–31]. *ZAP1* codes for a master zinc regulator in *C. gattii* and its is characterized in some fungal species [32]. Its absence was show to lead to reduction of anti-oxidative responses in *C. gattii* cells [33] as well in *S. cerevisiae* [29, 34].

RNA-Seq data from *C. gattii* R265 WT and a *zap1* mutant grown in YNB media with zinc deprivation were used to evaluate retrotransposon expression and silencing in stress conditions. The read counts of aligned libraries were quantified and the log2 fold change of differential expression analysis was quantified and normalized to Reads Per Kilobase of tanscript per Million mapped reads (RPKM) values for transcription measurement (Table 1, Additional file 16). No drastic changes in the RPKM values or the number of mapped reads distribution could be detected for both conditions (Additional files 17 and 18). Despite the variability of expression among different retrotransposons, no differential expression of such elements between WT and zap1 strains could be detected. The only two exceptions were RTs R265.26 and R265.38, which are only detected in WT or *zap1* conditions, respectively. This suggests that the absence of Zap1 transcription factor in a zinc-limiting environment alters the activity of such retrotransposons. Thus, it is possible that motif recognition on the retrotransposon promoter or a genomic localization near to Zap-1 target genes may be the cause of expression decline.

**Table 1:** RPKM and log2 fold change values in retrotransposon sequences

Retro transposon	RPKM sRNA_WT	RPKM mRNA_WT	RPKM mRNA_ZAP1	Log2 fold change (mRNA) ZAP1xWT
R265.1	2212.17	165.12	48.09	−1.78
R265.10	2728.76	182.41	318.81	0.80
R265.11	8396.39	14638.41	15671.02	0.10
R265.12	13322.54	133.26	16.63	−3.00
R265.13	3991.29	2050.13	677.89	−1.60
R265.14	8884.66	80.78	70.59	−0.19
R265.15	1676.88	0	0	N/A
R265.16	19299.97	6745.21	11206.42	0.73
R265.17	808.77	0	27	N/A
R265.18	5015.68	107.47	125.22	0.22
R265.19	48521.82	29562.31	36487.05	0.30
R265.2	2923.34	360.06	125.86	−1.52
R265.20	2567.82	168.44	183.99	0.13
R265.21	4481.6	23880.77	25869.49	0.11
R265.22	45424.93	259.33	226.62	−0.19
R265.23	3496.62	28.71	25.09	−0.19
R265.24	1074.35	145.76	42.46	−1.78
R265.25	18917.41	9940.13	12131.12	0.29
R265.26	2075.06	511.17	0	N/A
R265.27	4526.67	103.71	30.21	−1.78
R265.28	1553.01	40.86	35.71	−0.19
R265.29	5431.2	496.81	186.06	−1.42
R265.3	19795.43	7875.53	9215.24	0.23
R265.30	9472.7	2621.35	52.66	−5.64
R265.31	5159.63	129824.4	90122.72	−0.53
R265.34	4506.72	0	0	N/A
R265.36	103818.7	10138.81	20509.48	1.02
R265.37	39301.07	5627.55	22949.74	2.03
R265.38	3792.86	0	35.2714	N/A
R265.4	4525.26	75.71	132.32	0.80
R265.5	24813.18	22065.62	31063.56	0.49
R265.6	37947.45	514.37	345.77	−0.57
R265.7	22013.22	17762.5	16862.24	−0.07
R265.8	3893.5	241.84	1704.8	2.82

**Fig. 5:**
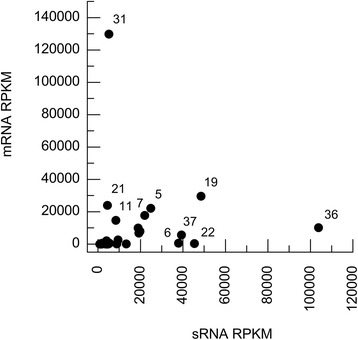
mRNA and sRNA retrotransposon correlation. RPKM values of *C. gattii* R265 retrotransposons for mRNA and sRNA expression of wild-type (WT) *C. gattii* R265 in a zinc deprivation culture

Retrotransposon propagation leads to genomic instability, and as a consequence, genetic diversity and phenotypic variation [35]. However, to avoid deleterious effects, there are several multiple transcriptional and posttranscriptional repressing mechanisms [36]. Among these mechanisms are those mediated by small RNAs, as previously described for the RNAi proficient *C. neoformans* H99 strain [9]. We therefore speculated that sRNAs could be controlling the expression of retrotransposons in *C. gattii* R265. We determined the sequence of small RNAs recovered form *C. gattii* R265 grow in zinc limitation conditions. Read length analysis revealed an abundance pattern somewhat similar to those observed in RNAi-proficient *C. neoformans* H99. While *C. gattii* most abundant reads ranges from 14 to 26 nt (data not shown), the majority of reads form *C. neoformans* library ranges from 21 to 23 nt [9]. The presence of high mRNA expression with low sRNA associated levels (R265.31, R265.21) and high sRNAs levels with low expressions of mRNAs (R265.36, R265.22, R265.37, R265.6) may indicate an sRNA-regulated silencing mechanism for retrotransposon propagation. The correlation of mRNA x sRNA RPKM values from WT libraries is exhibited in Fig. 5.

**Fig. 6:**
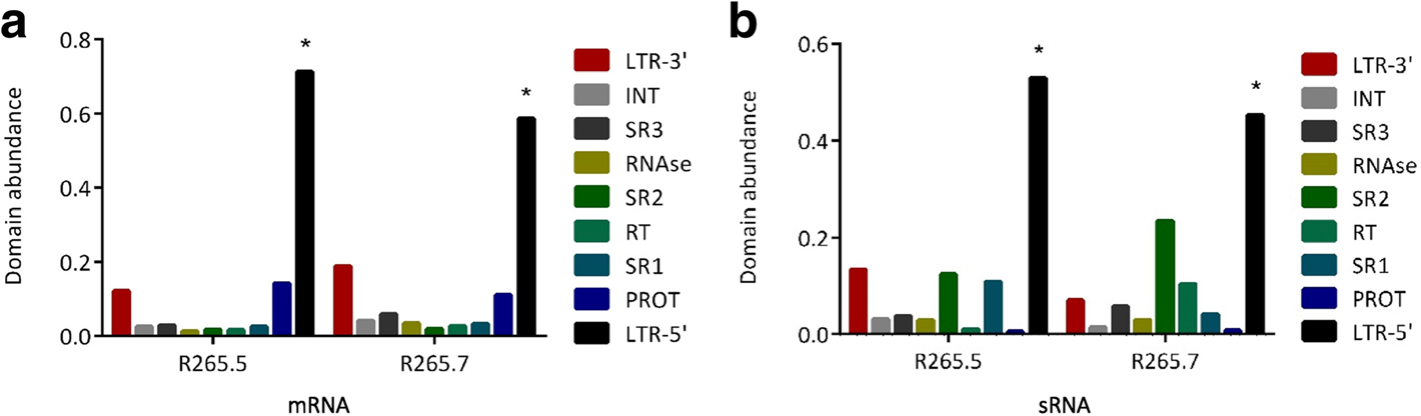
mRNA and sRNA reads profile mapping. Distribution of aligned reads from **a** mRNA and **b** sRNA libraries in terminal repeats, protein domains, and spacer regions inside retrotransposon sequences. Abbreviations: Long Terminal Repeats (LTR), Integrase (INT), Ribonuclease H (RNAse), Reverse Transcriptase (RT), Aspartyl Protease (PROT)

The mapping profile of sRNAs and mRNAs from the WT condition in the two most highly expressed full-length retrotransposons (R265.5 and R265.7) can be observed in Fig. 6. Domain loss in many sequences, as well as the mutations and frameshifts in coding regions that render several domains non-functional, hampers the correct evaluation of domain expression patterns for all retrotransposons. Visually, sRNAs seem to be widely distributed and mRNAs are more abundant in LTR-5′ (Additional files 19, 20 and 21). Statistically, the comparison between read counts of mRNAs distributed in LTRs, protein domains, and spacer regions (SR) exhibits significant a difference (*p* < 0,0001 to *p* < 0,05) due to the increased values of LTR-5′ read counts compared to other regions. As previously noted, long terminal repeats (LTRs) are important for transcription regulation (LTR-5′, specifically, contains the promoter sequence) and usually contain high amounts of aligned reads. The absence of statistical variation inside the internal region of retrotransposons (consistent with the entire retrotransposon transcription) and the higher density of RNA-Seq reads mapping to LTR-5′ was previously observed [37] in a large-scale LTR retrotransposon analysis of transcriptome data.

The sRNA profile analysis of raw read counts with R265.5 and R265.7 sequences reveals a decrease in aligned reads to protein domain sequences, especially reverse transcriptase (RT) and protease (PROT). As reported by Domingues and colleagues [38], most LTR retrotransposon families do not have high sRNA counts within the coding domains. The increased sRNAs read count in both LTRs (LTR-5′ with statistical significance) and spacer regions (SR1, SR2, and SR3) shows the possible target regions for sRNA activity in a silencing mechanism.

## Discussion

Retrotransposons are ubiquitously distributed in fungal species, albeit their structure is not conserved. The *pol* gene encodes a polyprotein composed of an aspartic protease, a reverse transcriptase, a ribonuclease H, and an integrase; the order and number of domains may differ between retrotransposon families, and most of the elements have incomplete *pol* genes. In addition, retrotransposons are generally characterized by the presence of LTR [22]. As previously described [2], *C. gattii* retrotransposon sequences can be grouped into (i) LTRs with associated, but incomplete, internal regions; and (ii) partial internal regions with no identified LTRs.

The comparative analysis of *C. gattii* retrotransposons showed here revealed a large diversity of retrotransposons among diverse genotypes. It is noteworthy that the number of retrotransposons families number changes in a genotype-associated fashion. The VGI genotype strains possess the higher detectable retrotransposons and consequently the higher density compared to strains from VGII, VGIII and VGIV genotypes. This diversification of the number of retrotransposon families among different fungal strains is in agreement with previous reports comparing the prevalence of such mobile elements among closely related species and strains of *Coccidioides immitis* and *Coccidioides posadasii* [39], as well for *Paracoccidioides* spp. [40].

The genomic sequence of 16 *C. gattii* strains are available and their analysis described. Comparative genomic analysis revealed that chromosomal structure was very conserved among the four varieties (VGI, VGII, VGIII, and VGIV). Furthermore, this conservation is even more pronounced among VGII strains [15]. As expected, our retrotransposon conservation analysis revealed the majority of these mobile elements present in R265 strain have orthologs in other *C. gattii* strains, most of them displaying also synteny.

Strains of the VGII genotype do not have a functional RNAi pathway, a highly conserved mechanism to control and inhibit retrotransposon expression through a homology-dependent gene-silencing complex [16]. It is therefore feasible to assume that VGII strains do not have an active RNAi pathway and that increased retrotransposons numbers are a consequence of increased retrotransposon expression and mobility, as seen in RNAi-deficient mutant of *C. neoformans* [8]. Unexpectedly, VGII strains displayed a smaller number of retrotransposon sequences compared to RNAi-proficient cryptococcal genotypes. Although R265 hypervirulence seems related to the absence of this regulatory mechanism, this observation suggests the existence of alternative control pathways or non-canonical Argonaute proteins, or the possibility that *C. gattii* (and not only VGII) has a more efficient (RNAi independent) system to control mobile element propagation. A predominance of LTR and DNA elements, LINEs, and low-complexity repeats in certain VGI strains was observed in *C. gattii* genotype analysis [15]. Furthermore, *AGO* gene absence in R265 and other VGII strains was not associated with gaps in the R265 genome [24]. However, it is unclear how far the incomplete assembly of genomes may alter the retrotransposon count. WM276 and NT10 have more retrotransposons and a well-assembled genome (WM276). Nevertheless, EJB2, E566, RU294, as both VGIII and VGIV strains, have RNAi pathways and exhibit similar sequence counts. The same explanation was suggested by Muszewska and colleagues [22] when analyzing differences in retrotransposon amounts between distinct strains of two *Coccidioides* species. Their findings further support that identified element amounts correlate with genomic sequences assembly lengths.

The retrotransposon expression analysis showed here revealed that all retrotransposons have detectable transcripts in distinct stress conditions. An uneven distribution of reads was detected among the *C. gattii* R265 transposons, with the large majority of reads matching one of the LTRs. This is in accordance to RNA-Seq assays using mRNA isolated from fission yeast [37].

To further evaluate retrotransposons expression, we performed a comparative transcriptome analysis using RNA-Seq data from *C. gattii* WT and *zap1* mutant strains cultured under zinc restriction [33]. Iron, copper, and zinc are essential metals for many processes in fungal pathogens. Zinc, specifically, is involved in transcriptional control, ROS detoxification, carbohydrate oxidation, and alcoholic fermentation [41, 42]. Thereby, in *Cryptococcus*, the ZIP family of plasma membrane transporters mediates zinc acquisition, especially in zinc-limiting conditions. The expression regulation of these transporter-encoding genes is performed by the transcriptional factor *ZAP1*. Our group previously demonstrated a severe impairment in ROS detoxification in cryptococcal cells lacking *ZAP1* gene [33]. This can be explained by the absence of indirect activation of many biological processes affected by zinc depletion in the *zap1* mutant, since the complete removal of this metal harms all cellular functions of zinc-dependent metalloproteins. In this way, the comparison of WT and *zap1* mutant cells allowed us to explore two distinct stress related conditions. However, our comparative analysis allowed the identification of only 2 stress-regulated retrotransposons.

Control of retrotransposon expression in fungal species is poorly characterized. Heterochromatin formation by H3mK9 histone methyltransferase recruitment in *Schizosaccharomyces pombe* is mediated by specific siRNAs acting in an Argonaute complex [43, 44]. *Saccharomyces cerevisiae* (which lacks the RNAi machinery for silencing mobile elements) produces antisense non-coding RNAs to regulate gene expression at the transcriptional level [26] and post-transcriptionally, as an auxiliary in copy number control mechanism. This maintains lower levels of mature integrase and reverse transcriptase, coupled with a truncated gag [45]. At the transcriptional level, Ty1 antisense RNAs regulate Ty1 retrotransposon expression by alterations in chromatin function [45].

In *C. neoformans*, retrotransposon activity is controlled by the RNAi pathway [8]. Stalled spliceosomes associate with a complex named SCANR, leading to the formation of siRNAs responsible for retrotransposon silencing via AGO dependent RNA degradation [9]. As *C. gattii* VGII strains lack components of RNAi pathway, it is reasonable to assume that this strategy is not used by VGII strains to control retrotransposons activity. However, the detection of sRNAs that map to retrotransposon sequences with low expression claims that additional mechanisms for retrotransposon activity control may exist in *C. gattii* VGII strains. It is noteworthy that *C. gattii* R265 possess a functional *DCR2* gene, which codes the enzyme responsible for the processing of siRNA precursors. In *C. neoformans,* Dcr2 was shown to be important for RNAi-mediated gene silencing [8]. In addition, the *C. gattii* VGII genomes accumulate a large amount of lost genes [16] and a hypervirulent phenotype. The absence of a canonical RNAi pathway can be a major influence on this profile. Nevertheless, the production of sRNAs means that *C. gattii* R265 has a secondary, alternative retrotransposon silencing mechanism, which guarantees genetic variation without the deleterious invasion of parasitic elements.

## Conclusions

WM276 (VGI) is the only strain with a well-assembled genome and is therefore used as a parameter for predicting results in *C. gattii* analyses. Differences in retrotransposon count can be explained by incomplete genome assemblies. This is mainly because VGIII and VGIV strains, which are genetically more similar to VGI, have a functional RNAi pathway, but also have low numbers of retrotransposons (as VGII) when compared to WM276 and NT10. Sequences grouped in TE clusters are a strong feature of WM276 retrotransposons. Retrotransposon orthology showed the prevalence of shared sequences with related strains, a result of “ancient” retrotransposons from before *C. gattii* diversification. The unique sequences of WM276, if not a genomic assembly consequence, may indicate recent transposition events. Synteny conservation reflects RNAi importance in the control of mobile element propagation. VGI molecular type has superior rates of synteny maintenance within genotypes than VGII, and the same number of retrotransposon-associated synteny block breakpoints. This confirms that R265 retrotransposons exert an effect on genome stability. Besides differences in TE content, the phylogenetic inference of the R265.19 element demonstrated that retrotransposon propagation by nucleotide substitution analysis resembles *C. gattii* strain expansion, also showing a greater conservation of RNase and reverse transcriptase in VGII. In addition, expression evaluation confirms the existence of retrotransposons expression regulation.

Further studies are necessary to fully identify the mobile elements in *C. gattii* and, especially, to completely assemble its genome(s). However, the presence of sRNAs as possible agents to control sequence mobilization creates a new basis for research on alternative pathways, *AGO-like* domains, and other mechanisms that promote non-canonical retrotransposon silencing in this medically relevant fungus.

## Methods

### Retrotransposon mining

Retrotransposon mining with LTR_Finder v1.0.6 [46], LTR_Harvest v1.5.7 [47], LTR_STRUC v1.1 [48], LTR_Seq [49], MGEScan v2 [50], and Transposon_PSI (http://transposonpsi.sourceforge.net) programs were used in *C. gattii* genomes with default parameters. The 17 *C. gattii* genomes (Table 2) from VGI, VGII, VGIII, and VGIV molecular types used in this work are available in the NCBI database .

**Table 2:** *C. gattii* strains used in retrotransposon mining

Molecular type	Strain	NCBI Reference number
VGI	WM276	GCA_000185945.1
VGI	NT10	GCA_000935105.1
VGI	E566	GCA_000875815.1
VGI	EJB2	GCA_000835745.1
VGI	RU294	GCA_000836355.1
VGII	99473	GCA_000836455.1
VGII	2001935	GCA_000835815.1
VGII	CA1014	GCA_000875795.1
VGII	CBS7750	GCA_000499585.1
VGII	CBS10090	GCA_000835765.1
VGII	LA55	GCA_000836315.1
VGII	MMRL2647	GCA_000875855.1
VGII	R265	GCA_000149475.3
VGII	RAM5	GCA_000836375.1
VGIII	CA1280	GCA_000836335.1
VGIII	CA1873	GCA_000855695.1
VGIV	IND107	GCA_000835755.1

New sequences were discovered by searching with the Blastn algorithm v2.2.31 (megablast) [51, 52] of known retrotransposons against genomes. Sequence confirmation by retrotransposon domain presence (gag, integrase, reverse transcriptase, RNase H, and aspartic protease) was evaluated using Blast Conserved Domains [53].

### Retrotransposon orthology between genomes

Sequences that are putatively orthologous to R265 and WM276 retrotransposons were inferred by *best bi-directional hits* (BBH - with minimum 90% query cover, 90% identity) in a Blastn search (megablast) with retrotransposon sequences against genomes. R265 retrotransposons distributed in all *C. gattii* strains (R265.3, R265.19, and R265.22) were tested for orthologous sequences in *C. neoformans* H99 (GCA_000149245.3) by the same methodology.

### Synteny evaluation

Multiple genome alignment for synteny analysis of widely distributed R265.3, R265.19, and R265.22 orthologous sequences was performed in Mauve aligner v20150226 [54] with default parameters for all 17 *C. gattii* strains. Intrinsic synteny by orthologous retrotransposon localization inside VGI and VGII genotypes was analyzed on an independent multiple genome alignment of VGI and VGII strains. Syntenic blocks between genomes were represented by the same color.

### Phylogenetic inference

Reverse trancriptase and RNase H domain sequences (nucleotide) from R265.19 and orthologous retrotransposons (17 *C. gattii* sequences and one *C. neoformans* H99 orthologue, as an outgroup) were separately aligned using MAFFT v7 [55] with default parameters for alignment strategy and adjusted sequence direction option. jModelTest v2.1.10 [56] was utilized to determine the best evolutionary model describing the data and Mr. Bayes v3.2.6 [57] was used for Bayesian analysis of phylogenetic inference. Computed trees were visualized with FigTree v1.4.2 (http://tree.bio.ed.ac.uk/software/figtree/) .

### *C. gattii* R265 RNA-Seq of small RNAs

The *C. gattii* strain R265 was cultured in YPD media (2% glucose; 2% peptone; 1% yeast extract) for 18 h at 200 rpm and 30 °C. Next, the cells were centrifuged (5000 × *g* – 5 min) and washed with PBS (NaCl 137 mM; KCl 2,7 mM; Na_2_HPO_4_ 10 mM; KH_2_PO_4_ 1,8 mM; pH 7,4). The cell pellet was resuspended in 20 mL of 1x YNB (*Yeast Nitrogen Base*) and diluted to 10^7^ cells (OD_600_ = 1). For the zinc deprivation assay, the quantified cells were inoculated in 100 mL of YNB 1x + 10 μM TPEN and kept for 18 h at 200 rpm. Afterwards, the cells were centrifuged (5000 g–5 min), frozen in liquid nitrogen, and placed in an ultrafreezer (−80 ° C) for lyophilization.

RNA extraction was performed with Trizol® (Invitrogen) reagent according to the manufacturer’s protocol. Sample quantification was evaluated by fluorometric analysis with Qubit and Quant-It (Invitrogen), and samples were stored in RNA stable reagent (Biometrica). sRNAs were purified from total RNA samples, processed, and sequenced by Fasteris Life Sciences SA, Plan-les-Ouates, Switzerland using Solexa technology on an Illumina Hi Seq 2000 platform.

### sRNAs prediction

Reads from the zinc deprivation were filtered. Sequences from ribosomal RNAs (rRNAs), transporter RNAs (tRNAs), small nuclear RNAs (snRNAs), and small nucleolar RNAs (snoRNAs) present in the Rfam data bank [58] were excluded. Only sequences between 12 and 28 nucleotides were retained in the library. These sequences were aligned to Retrotransposon sequences using Bowtie2 [59]. Domains containing values of RPKM equal or larger than 500 RPKM were considered as possible sRNA targets.

### Expression evaluation

The *C. gattii* R265 mRNA libraries of WT and *ZAP1* depleted mutant (both grown in zinc deprivation conditions) used in this study were obtained from Schneider and colleagues [33].

RNA-Seq libraries (mRNAS and sRNAs) were aligned against the *C. gattii* R265 genome with the Tophat v2.1.1 program [60] unique alignment option (*max-multihits = 1*). Mapped reads were visualized with IGV v2.3.78 [61] and quantified with Samtools v0.1.19 [62]. RPKM (*reads Per kilobase per million*) values were manually calculated and Log2 fold change was determined using the DESeq2 package of R [63]. Domain abundance inside retrotransposons was quantified by the division of raw read counts from each region by the sequence total read count. The statistical analysis of results was performed by ANOVA.

## Additional files

Additional file 1: Retrotransposon sequence list from all 17 *C. gattii* strains. The retrotransposon list contains detailed descriptions of molecular type, *C. gattii* strain, NCBI reference for supercontig, sequence coordinates, LTR retrotransposon family and domains; as well as, the annotation of orthologous sequences in WM276 and R265 strains. The synteny analysis columns indicate presence (“YES”) and absence (“NO”) of synteny; and “NA” to not analyzed data. (XLS 118 kb)

Additional file 2: C. gattii multiple genome alignment. Multiple genome alignment of VGI, VGII, VGIII and VGIV molecular types with Mauve aligner. Syntenic blocks shared by genomes are represented with the same color and are connected by lines; red lines indicate chromosome or supercontig boundaries. (JPG 592 kb)

Additional file 3: VGI multiple genome alignment. Multiple genome alignment of strains from VGI molecular type with Mauve aligner. Syntenic blocks shared between genomes are represented with the same color and are connected by lines; red lines indicate chromosome or supercontig boundaries. (JPG 1033 kb)

Additional file 4: Retrotransposon-associated synteny breakpoints 1 in WM276. Figure shows a multiple genome alignment of VGI strains. Syntenic blocks shared by genomes are represented with the same color and are connected by lines; red lines indicate chromosome or supercontig boundaries. The location of associated retrotransposons WM276.1, WM276.2, WM276.3 and WM276.4 in chromosome A of WM276 strain is represented by the black rectangle. Microsynteny disruption by retrotransposon insertion can be observed as white spaces inside blocks. (JPG 308 kb)

Additional file 5: Retrotransposon-associated synteny breakpoints 2 in WM276. Figure shows a multiple genome alignment of VGI strains. Syntenic blocks shared by genomes are represented with the same color and are connected by lines; red lines indicate chromosome or supercontig boundaries. The location of associated retrotransposons WM276.26, WM276.75, WM276.27 and WM276.28 in chromosome E of WM276 strain is represented by the black rectangle. Microsynteny disruption by retrotransposon insertion can be observed as white spaces inside blocks. (JPG 304 kb)

Additional file 6: Retrotransposon-associated synteny breakpoints 3 in WM276. Figure shows a multiple genome alignment of VGI strains. Syntenic blocks shared by genomes are represented with the same color and are connected by lines; red lines indicate chromosome or supercontig boundaries. The location of associated retrotransposons WM276.44, WM276.46 and WM276.47 in chromosome H of WM276 strain is represented by the black rectangle. Microsynteny disruption by retrotransposon insertion can be observed as white spaces inside blocks. (JPG 328 kb)

Additional file 7: Retrotransposon-associated synteny breakpoints 4 in WM276. Figure shows a multiple genome alignment of VGI strains. Syntenic blocks shared by genomes are represented with the same color and are connected by lines; red lines indicate chromosome or supercontig boundaries. The location of associated retrotransposons WM276.52, WM276.53, WM276.54 and WM276.55 in chromosome J of WM276 strain is represented by the black rectangle. Microsynteny disruption by retrotransposon insertion can be observed as white spaces inside blocks. (JPG 356 kb)

Additional file 8: Retrotransposon-associated synteny breakpoints 5 in WM276. Figure shows a multiple genome alignment of VGI strains. Syntenic blocks shared by genomes are represented with the same color and are connected by lines; red lines indicate chromosome or supercontig boundaries. The location of associated retrotransposons WM276.72 and WM276.73 in chromosome N of WM276 strain is represented by the black rectangle. Microsynteny disruption by retrotransposon insertion can be observed as white spaces inside blocks. (JPG 329 kb)

Additional file 9: Retrotransposon-associated synteny breakpoints 1 in R265. Figure shows a multiple genome alignment of VGII strains. Syntenic blocks shared by genomes are represented with the same color and are connected by lines; red lines indicate chromosome or supercontig boundaries. The location of associated retrotransposons R265.12 and R265.13 in supercontig 7 of R265 strain is represented by the black rectangle. Microsynteny disruption by retrotransposon insertion can be observed as white spaces inside blocks. (JPG 483 kb)

Additional file 10: Retrotransposon-associated synteny breakpoints 2 in R265. Figure shows a multiple genome alignment of VGII strains. Syntenic blocks shared by genomes are represented with the same color and are connected by lines; red lines indicate chromosome or supercontig boundaries. The location of associated retrotransposons R265.29, R265.30 and R265.31 in supercontig 25 of R265 strain is represented by the black rectangle. Microsynteny disruption by retrotransposon insertion can be observed as white spaces inside blocks. (JPG 436 kb)

Additional file 11: Retrotransposon-associated synteny breakpoints 3 in R265. Figure shows a multiple genome alignment of VGII strains. Syntenic blocks shared by genomes are represented with the same color and are connected by lines; red lines indicate chromosome or supercontig boundaries. The location of associated retrotransposons R265.23 and R265.24 in supercontig 17 of R265 strain is represented by the black rectangle. Microsynteny disruption by retrotransposon insertion can be observed as white spaces inside blocks. (JPG 493 kb)

Additional file 12: Retrotransposon-associated synteny breakpoints 4 in R265. Figure shows a multiple genome alignment of VGII strains. Syntenic blocks shared by genomes are represented with the same color and are connected by lines; red lines indicate chromosome or supercontig boundaries. The location of associated retrotransposons R265.26, R265.27, R265.34 and R265.28 in supercontig 21 of R265 strain is represented by the black rectangle. Microsynteny disruption by retrotransposon insertion can be observed as white spaces inside blocks. (JPG 508 kb)

Additional file 13: Retrotransposon-associated synteny breakpoints 5 in R265. Figure shows a multiple genome alignment of VGII strains. Syntenic blocks shared by genomes are represented with the same color and are connected by lines; red lines indicate chromosome or supercontig boundaries. The location of associated retrotransposons R265.38 and R265.20 in supercontig 13 of R265 strain is represented by the black rectangle. Microsynteny disruption by retrotransposon insertion can be observed as white spaces inside blocks. (JPG 487 kb)

Additional file 14: VGII multiple genome alignment. Multiple genome alignment of strains from VGII molecular type with Mauve aligner. Syntenic blocks shared by genomes are represented with the same color and are connected by lines; red lines indicate chromosome or supercontig boundaries. (JPG 2507 kb)

Additional file 15: Synteny analysis of R265.3, R265.19 and R265.22 orthologous sequences. Localization of orthologous retrotransposons of R265.3, R265.19, and R265.22 sequences are indicated by black lines in a multiple genome alignment with strains of VGI, VGII, VGIII, and VGIV molecular types. Orthologous sequences in each strain are named by the retrotransposon number. Syntenic blocks shared by genomes are represented with the same color and are connected by lines; red lines indicate chromosome or supercontig boundaries. (JPG 1397 kb)

Additional file 16: Raw read counts of mRNAs and sRNAs. Raw counts of mapped reads from mRNA and sRNA libraries to *C. gattii* R265 retrotransposons by the TopHat aligner. (XLSX 10 kb)

Additional file 17: Read counts values of retrotransposon sequences. Raw read counts distribution of retrotransposon sequences in WT and ZAP1 conditions. Distribution values are shown in a log scale. (JPG 27 kb)

Additional file 18: RPKM values of retrotransposon sequences. RPKM distribution of retrotransposon sequences in WT and ZAP1 conditions. Distribution values are shown in a log scale. (JPG 31 kb)

Additional file 19: Reads mapping profile of in full-length retrotransposons. Visualization of mapped reads profile from mRNA and sRNA libraries in full-length retrotransposons R265.5 and R265.7. Abbreviations: Long Terminal Repeats (LTR), Integrase (INT), Ribonuclease H (RNAse), Reverse Transcriptase (RT), Aspartyl Protease (PROT). (JPG 289 kb)

Additional file 20: Reads mapping profile of highly expressed retrotransposons. Visualization of mapped reads profile from mRNA and sRNA libraries in highly expressed sequences R265.19 and R265.31. Abbreviations: Long Terminal Repeats (LTR), Integrase (INT), Ribonuclease H (RNAse), Reverse Transcriptase (RT), Aspartyl Protease (PROT). (JPG 285 kb)

Additional file 21: Reads mapping profile of highly repressed retrotransposons. Visualization of mapped reads profile from mRNA and sRNA libraries in highly repressed sequences R265.36 and R265.37. Abbreviations: Long Terminal Repeats (LTR), Integrase (INT), Ribonuclease H (RNAse), Reverse Transcriptase (RT), Aspartyl Protease (PROT). (JPG 287 kb)

## Abbreviations

dsRNA: Double stranded RNA
GAGs: Group-specific AntiGen
LTR: Long terminal repeats
OD_600_: Optical density at 600 nm
PBS: Phosphate buffered saline
RdRP: RNA dependent RNA polymerase
RISC: RNA-induced silencing complex
RNAi: Interfering RNA
ROS: Reactive oxygen species
RPKM: Reads Per Kilobase of tanscript per Million mapped reads
siRNA: Small interfering RNA
sRNA: Small RNA
TE: Transposable elements
TPEN: N,N,N′,N′-tetrakis(2-pyridinylmethyl)-1,2-ethanediamine
VG: Variety *gattii*
VLPs: Virus-Like Particles
WT: Wild type
YNB: Yeast nitrogen base
YPD: Yeast peptone dextrose
