# Supplementary figures and images for: sRNAs as possible regulators of retrotransposon activity in Cryptococcus gattii VGII
Source: BMC Genomics. 2017 Apr 12;18:294. doi: 10.1186/s12864-017-3688-4 (PMC5389150; doi:10.1186/s12864-017-3688-4)

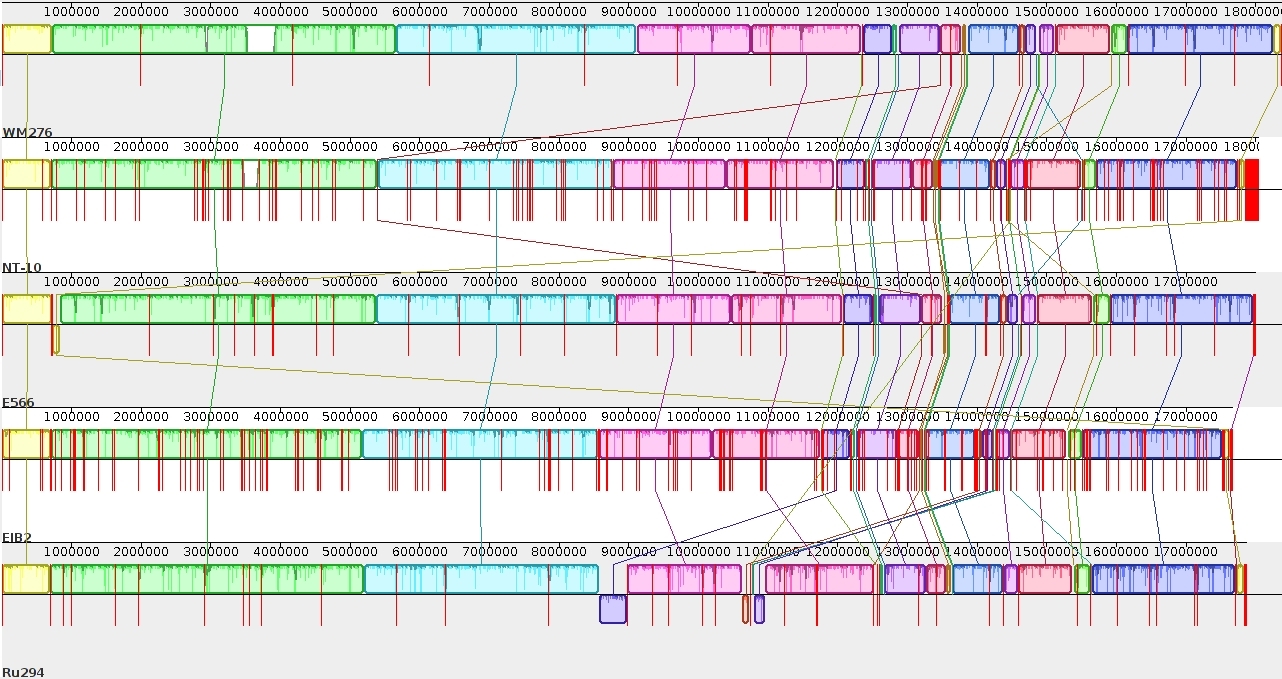

Supplement: Supplementary file 2 — C. gattii multiple genome alignment. Multiple genome alignment of VGI, VGII, VGIII and VGIV molecular types with Mauve aligner. Syntenic blocks shared by genomes are represented with the same color and are connected by lines; red lines indicate chromosome or supercontig boundaries. (JPG 592 kb) [file 12864_2017_3688_MOESM2_ESM.jpg]

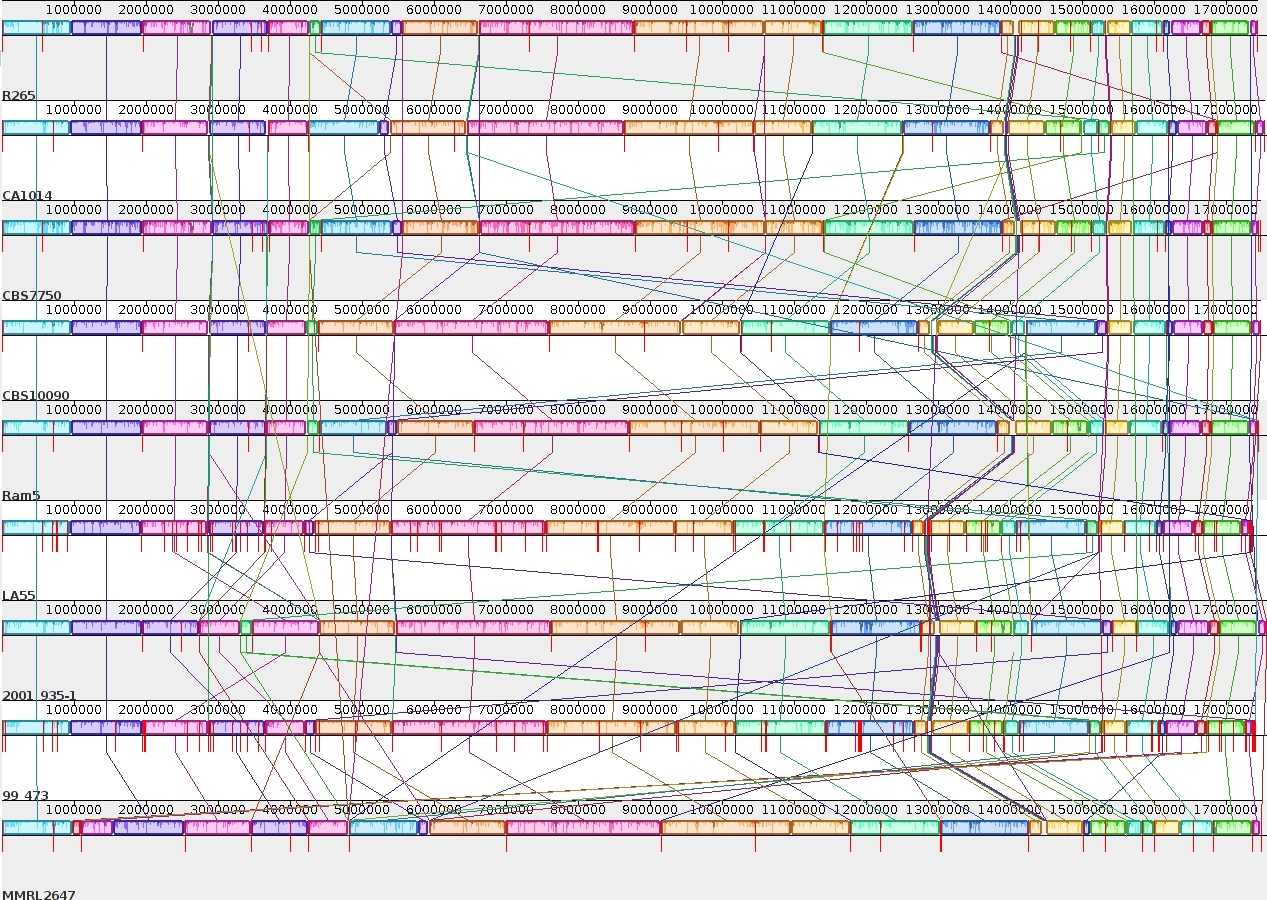

Supplement: Supplementary file 3 — VGI multiple genome alignment. Multiple genome alignment of strains from VGI molecular type with Mauve aligner. Syntenic blocks shared between genomes are represented with the same color and are connected by lines; red lines indicate chromosome or supercontig boundaries. (JPG 1033 kb) [file 12864_2017_3688_MOESM3_ESM.jpg]

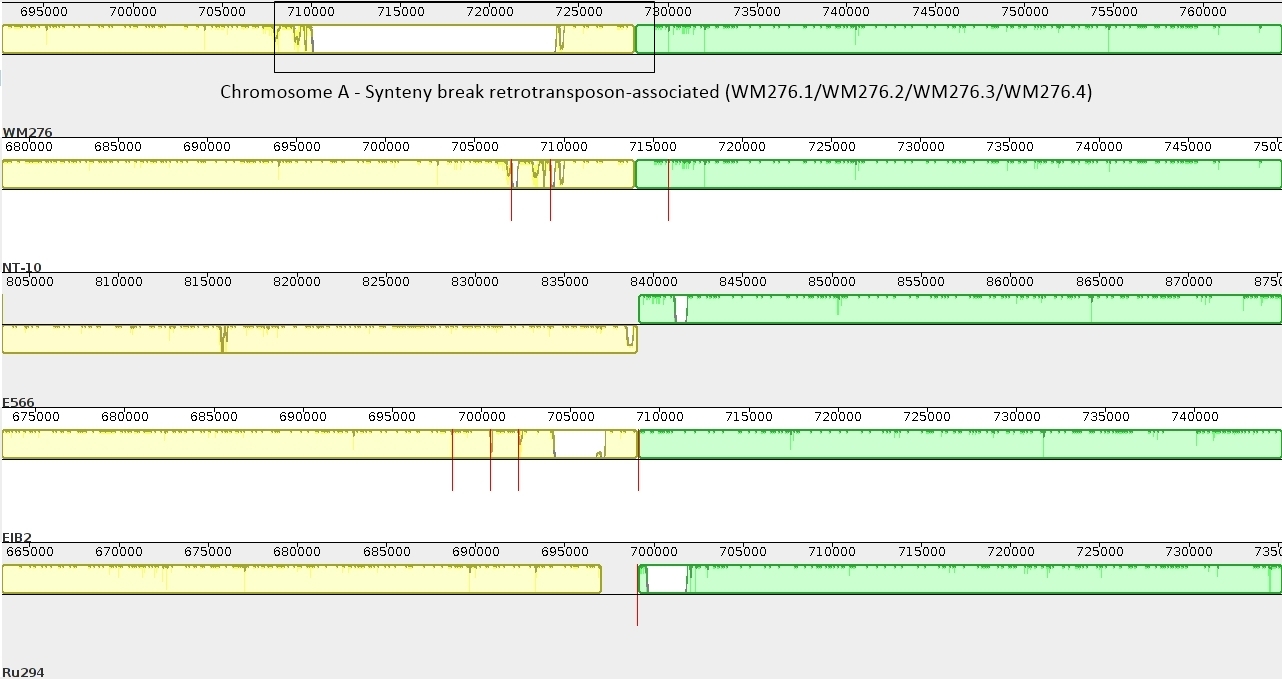

Supplement: Supplementary file 4 — Retrotransposon-associated synteny breakpoints 1 in WM276. Figure shows a multiple genome alignment of VGI strains. Syntenic blocks shared by genomes are represented with the same color and are connected by lines; red lines indicate chromosome or supercontig boundaries. The location of associated retrotransposons WM276.1, WM276.2, WM276.3 and WM276.4 in chromosome A of WM276 strain is represented by the black rectangle. Microsynteny disruption by retrotransposon insertion can be observed as white spaces inside blocks. (JPG 308 kb) [file 12864_2017_3688_MOESM4_ESM.jpg]

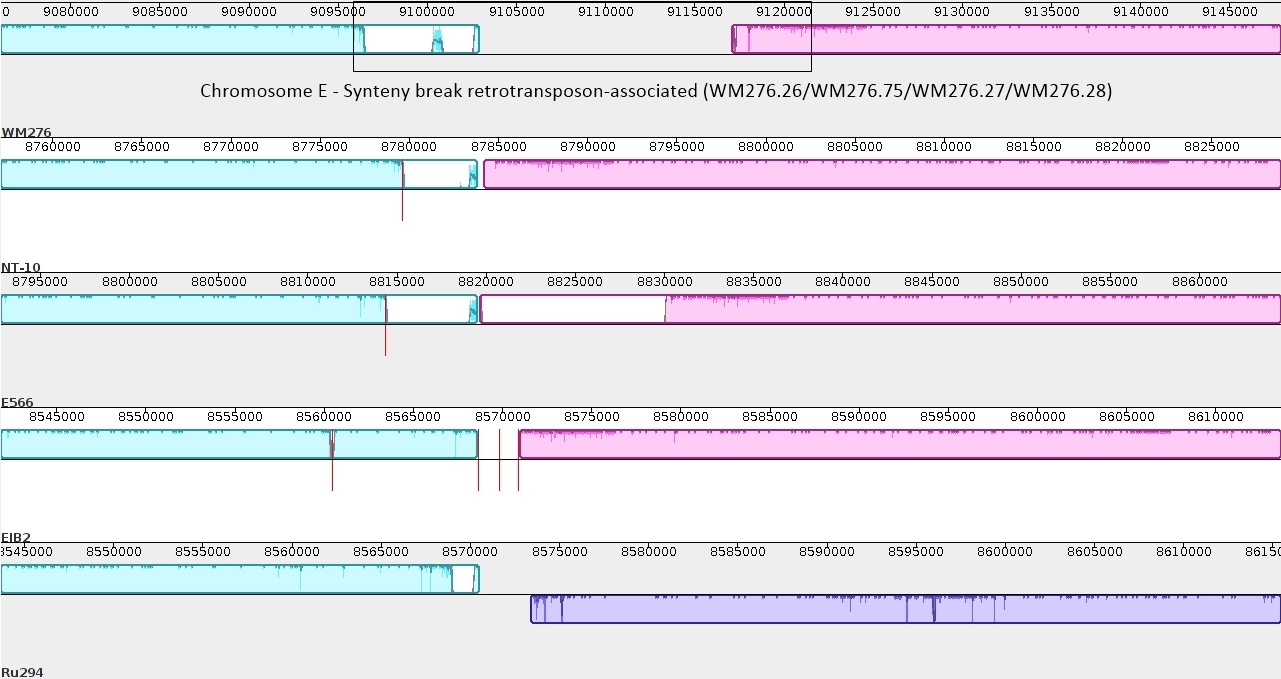

Supplement: Supplementary file 5 — Retrotransposon-associated synteny breakpoints 2 in WM276. Figure shows a multiple genome alignment of VGI strains. Syntenic blocks shared by genomes are represented with the same color and are connected by lines; red lines indicate chromosome or supercontig boundaries. The location of associated retrotransposons WM276.26, WM276.75, WM276.27 and WM276.28 in chromosome E of WM276 strain is represented by the black rectangle. Microsynteny disruption by retrotransposon insertion can be observed as white spaces inside blocks. (JPG 304 kb) [file 12864_2017_3688_MOESM5_ESM.jpg]

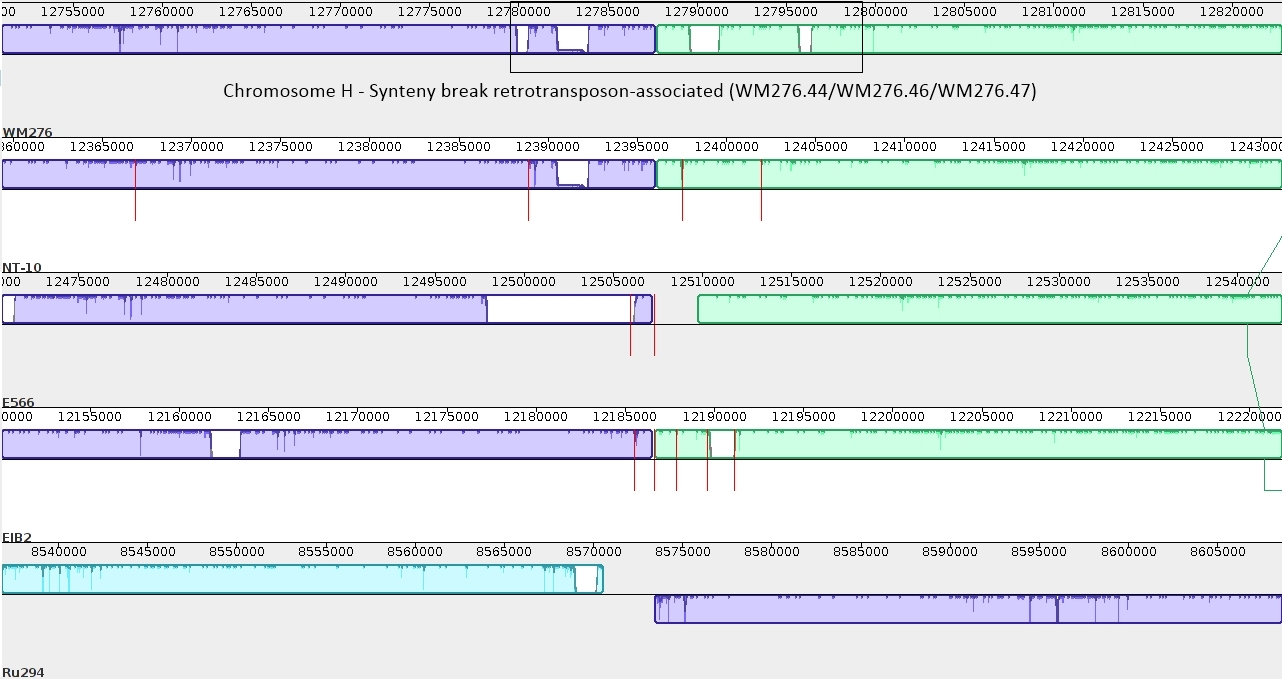

Supplement: Supplementary file 6 — Retrotransposon-associated synteny breakpoints 3 in WM276. Figure shows a multiple genome alignment of VGI strains. Syntenic blocks shared by genomes are represented with the same color and are connected by lines; red lines indicate chromosome or supercontig boundaries. The location of associated retrotransposons WM276.44, WM276.46 and WM276.47 in chromosome H of WM276 strain is represented by the black rectangle. Microsynteny disruption by retrotransposon insertion can be observed as white spaces inside blocks. (JPG 328 kb) [file 12864_2017_3688_MOESM6_ESM.jpg]

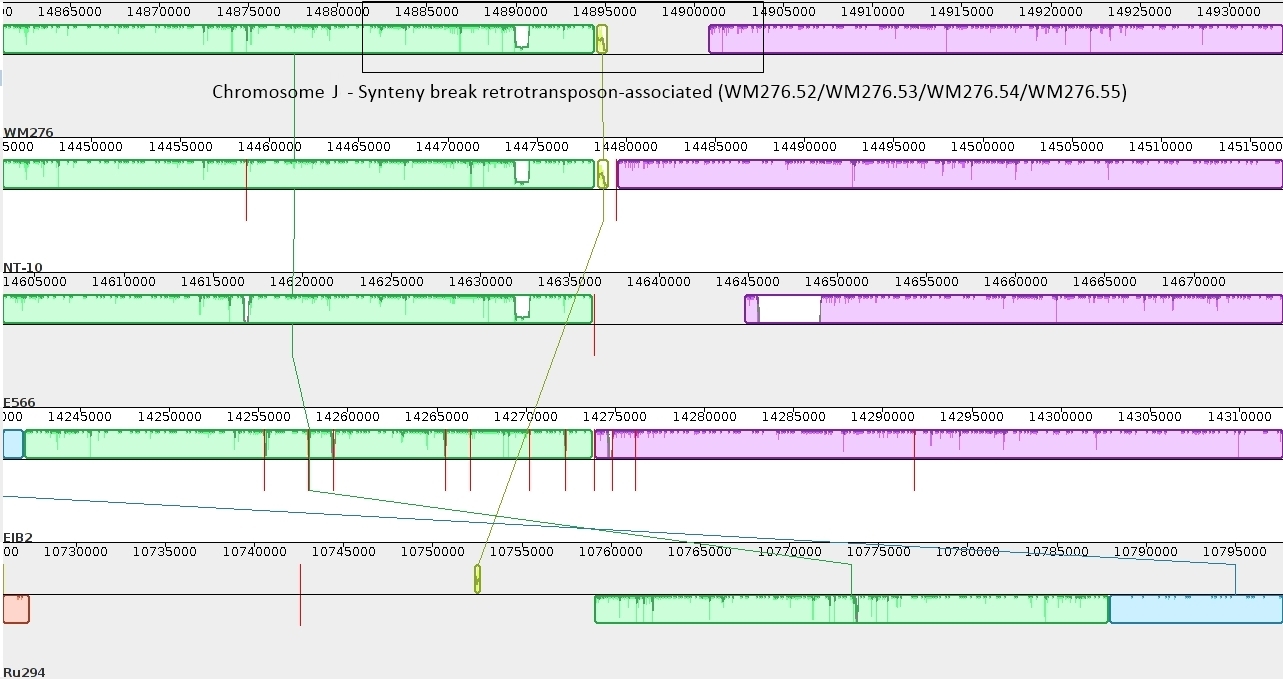

Supplement: Supplementary file 7 — Retrotransposon-associated synteny breakpoints 4 in WM276. Figure shows a multiple genome alignment of VGI strains. Syntenic blocks shared by genomes are represented with the same color and are connected by lines; red lines indicate chromosome or supercontig boundaries. The location of associated retrotransposons WM276.52, WM276.53, WM276.54 and WM276.55 in chromosome J of WM276 strain is represented by the black rectangle. Microsynteny disruption by retrotransposon insertion can be observed as white spaces inside blocks. (JPG 356 kb) [file 12864_2017_3688_MOESM7_ESM.jpg]

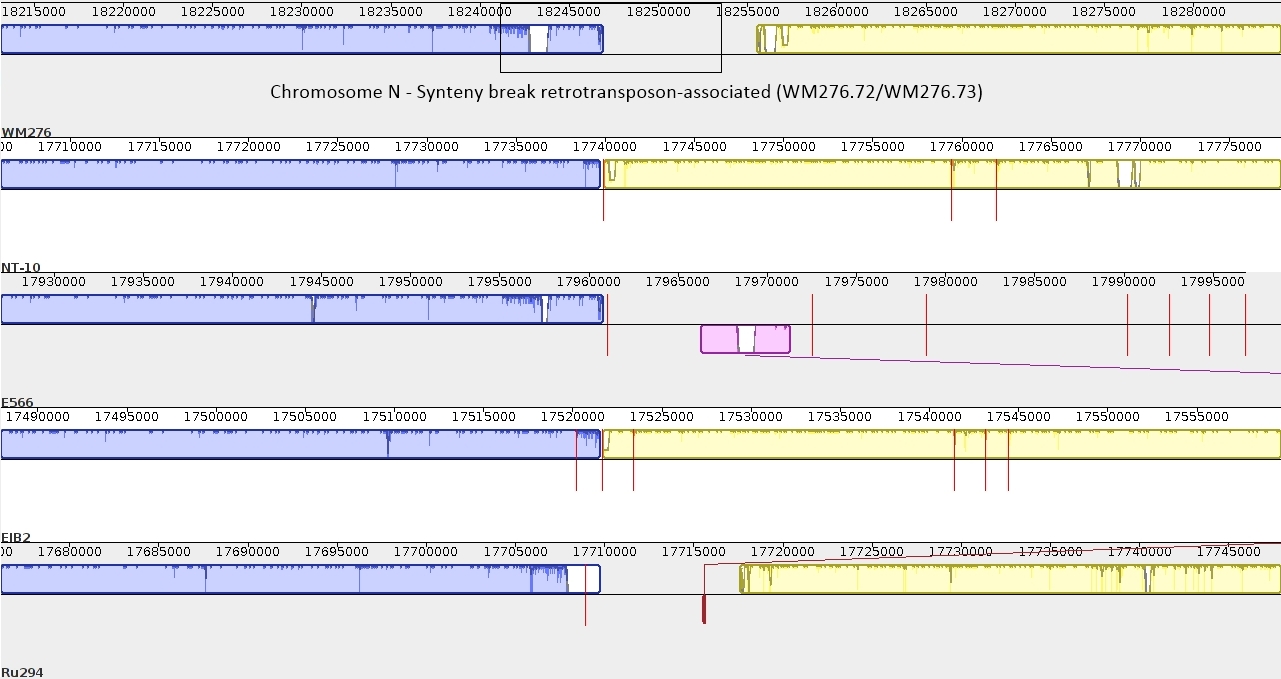

Supplement: Supplementary file 8 — Retrotransposon-associated synteny breakpoints 5 in WM276. Figure shows a multiple genome alignment of VGI strains. Syntenic blocks shared by genomes are represented with the same color and are connected by lines; red lines indicate chromosome or supercontig boundaries. The location of associated retrotransposons WM276.72 and WM276.73 in chromosome N of WM276 strain is represented by the black rectangle. Microsynteny disruption by retrotransposon insertion can be observed as white spaces inside blocks. (JPG 329 kb) [file 12864_2017_3688_MOESM8_ESM.jpg]

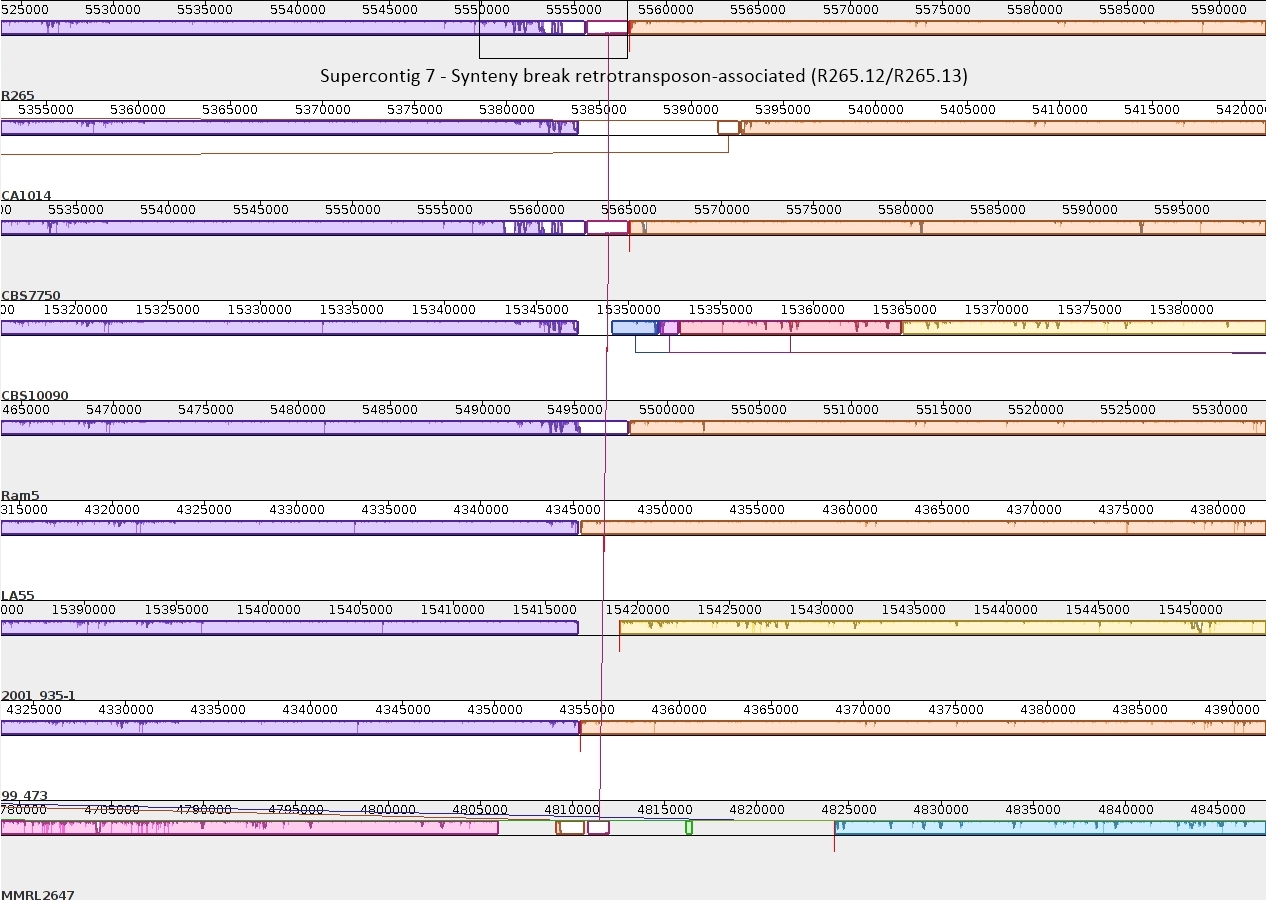

Supplement: Supplementary file 9 — Retrotransposon-associated synteny breakpoints 1 in R265. Figure shows a multiple genome alignment of VGII strains. Syntenic blocks shared by genomes are represented with the same color and are connected by lines; red lines indicate chromosome or supercontig boundaries. The location of associated retrotransposons R265.12 and R265.13 in supercontig 7 of R265 strain is represented by the black rectangle. Microsynteny disruption by retrotransposon insertion can be observed as white spaces inside blocks. (JPG 483 kb) [file 12864_2017_3688_MOESM9_ESM.jpg]

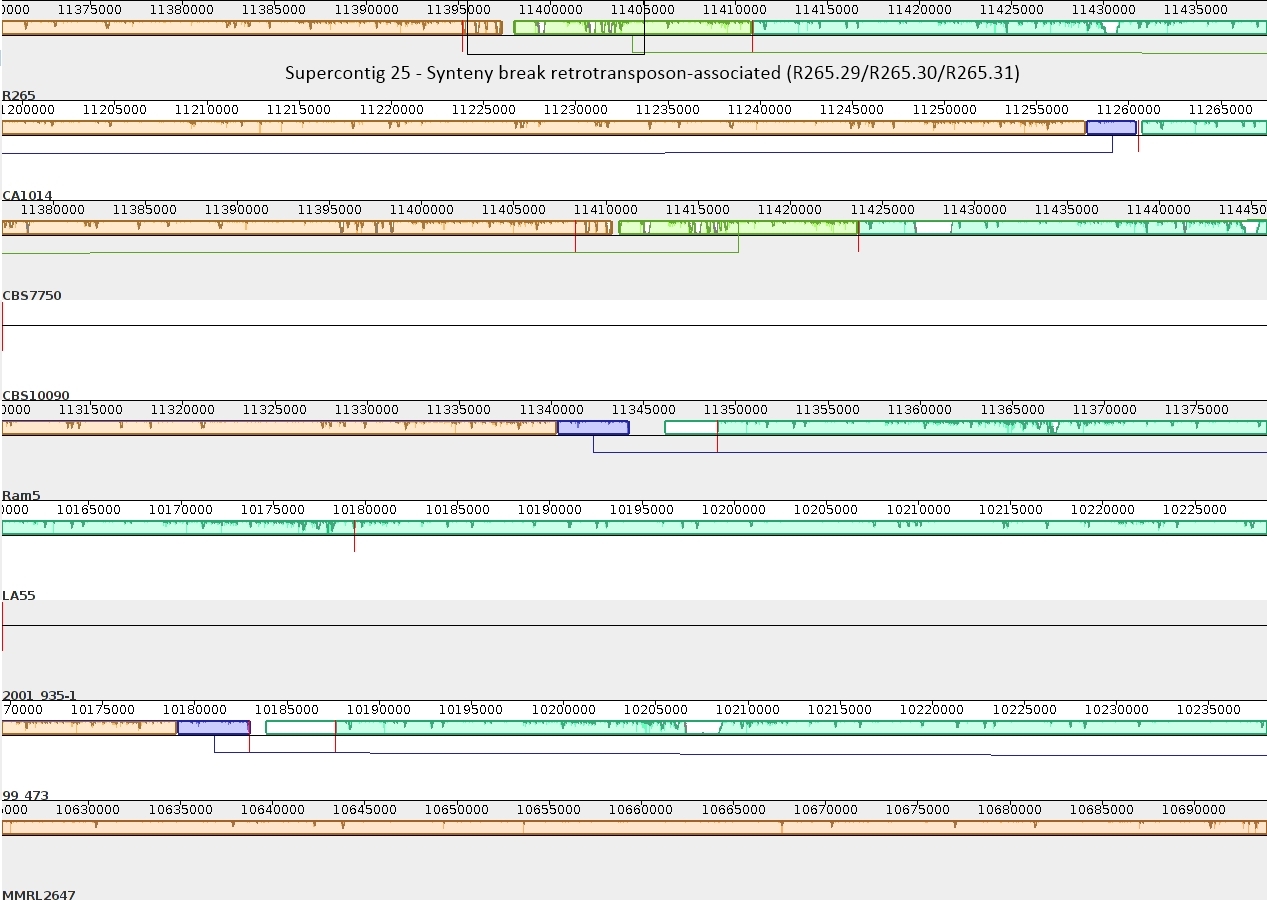

Supplement: Supplementary file 10 — Retrotransposon-associated synteny breakpoints 2 in R265. Figure shows a multiple genome alignment of VGII strains. Syntenic blocks shared by genomes are represented with the same color and are connected by lines; red lines indicate chromosome or supercontig boundaries. The location of associated retrotransposons R265.29, R265.30 and R265.31 in supercontig 25 of R265 strain is represented by the black rectangle. Microsynteny disruption by retrotransposon insertion can be observed as white spaces inside blocks. (JPG 436 kb) [file 12864_2017_3688_MOESM10_ESM.jpg]

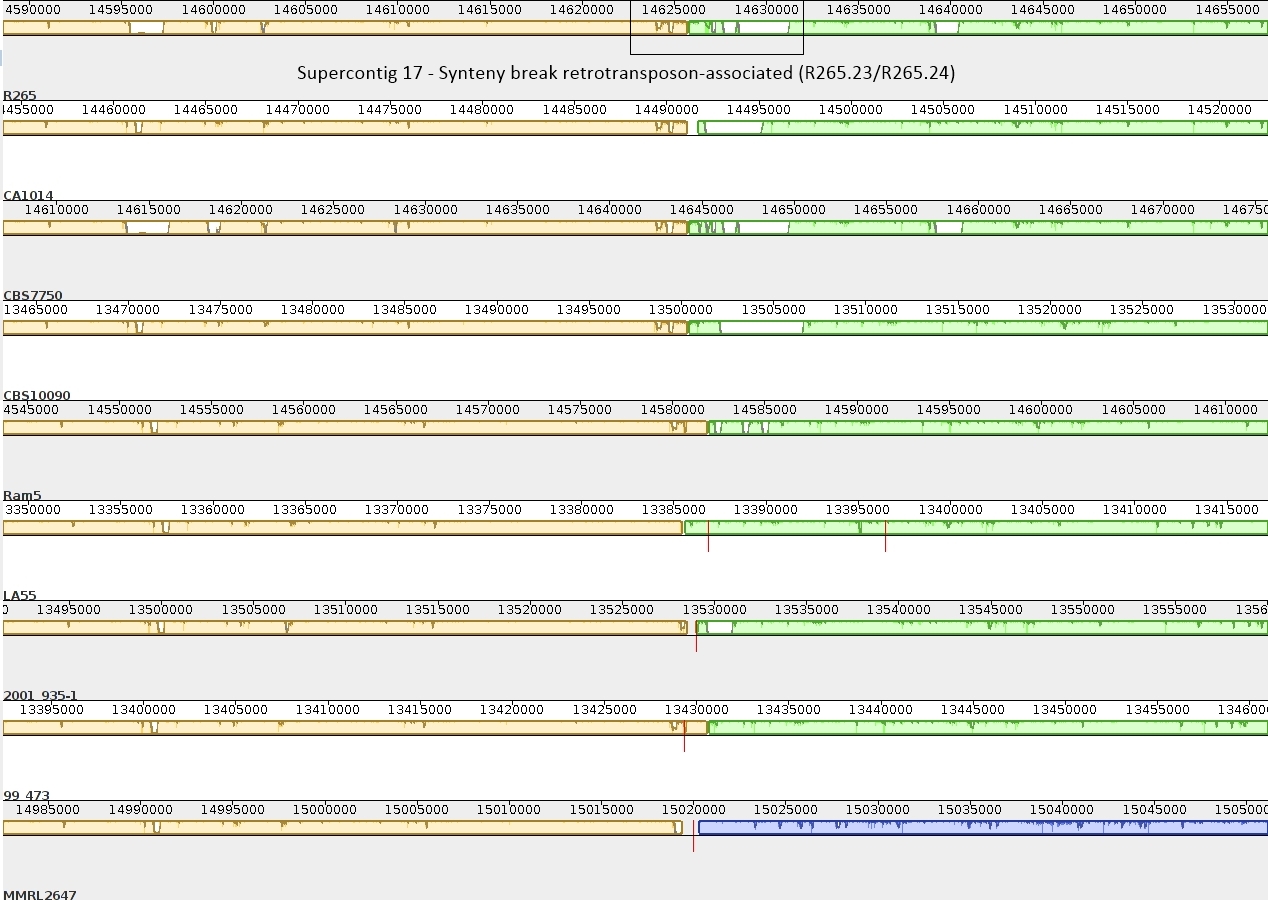

Supplement: Supplementary file 11 — Retrotransposon-associated synteny breakpoints 3 in R265. Figure shows a multiple genome alignment of VGII strains. Syntenic blocks shared by genomes are represented with the same color and are connected by lines; red lines indicate chromosome or supercontig boundaries. The location of associated retrotransposons R265.23 and R265.24 in supercontig 17 of R265 strain is represented by the black rectangle. Microsynteny disruption by retrotransposon insertion can be observed as white spaces inside blocks. (JPG 493 kb) [file 12864_2017_3688_MOESM11_ESM.jpg]

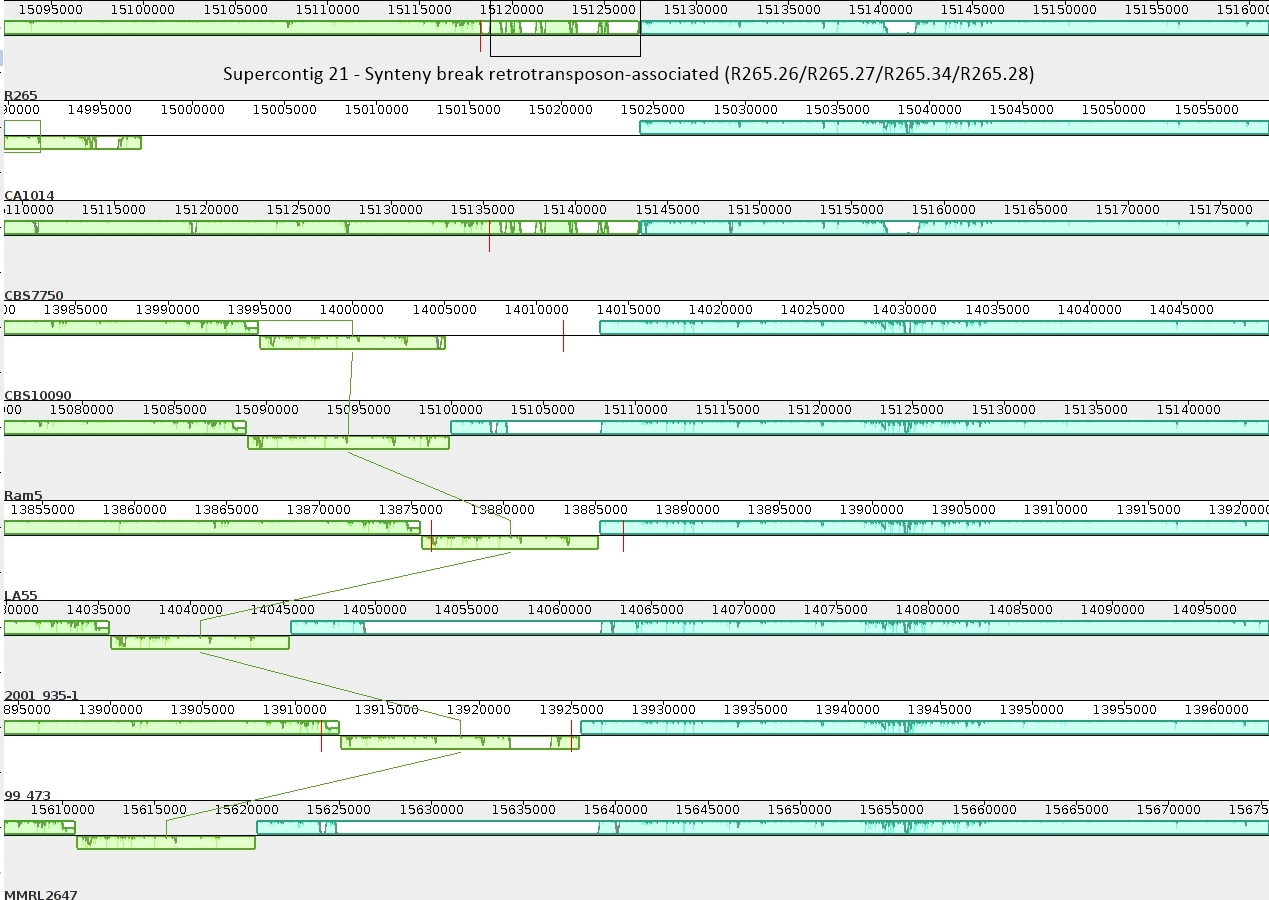

Supplement: Supplementary file 12 — Retrotransposon-associated synteny breakpoints 4 in R265. Figure shows a multiple genome alignment of VGII strains. Syntenic blocks shared by genomes are represented with the same color and are connected by lines; red lines indicate chromosome or supercontig boundaries. The location of associated retrotransposons R265.26, R265.27, R265.34 and R265.28 in supercontig 21 of R265 strain is represented by the black rectangle. Microsynteny disruption by retrotransposon insertion can be observed as white spaces inside blocks. (JPG 508 kb) [file 12864_2017_3688_MOESM12_ESM.jpg]

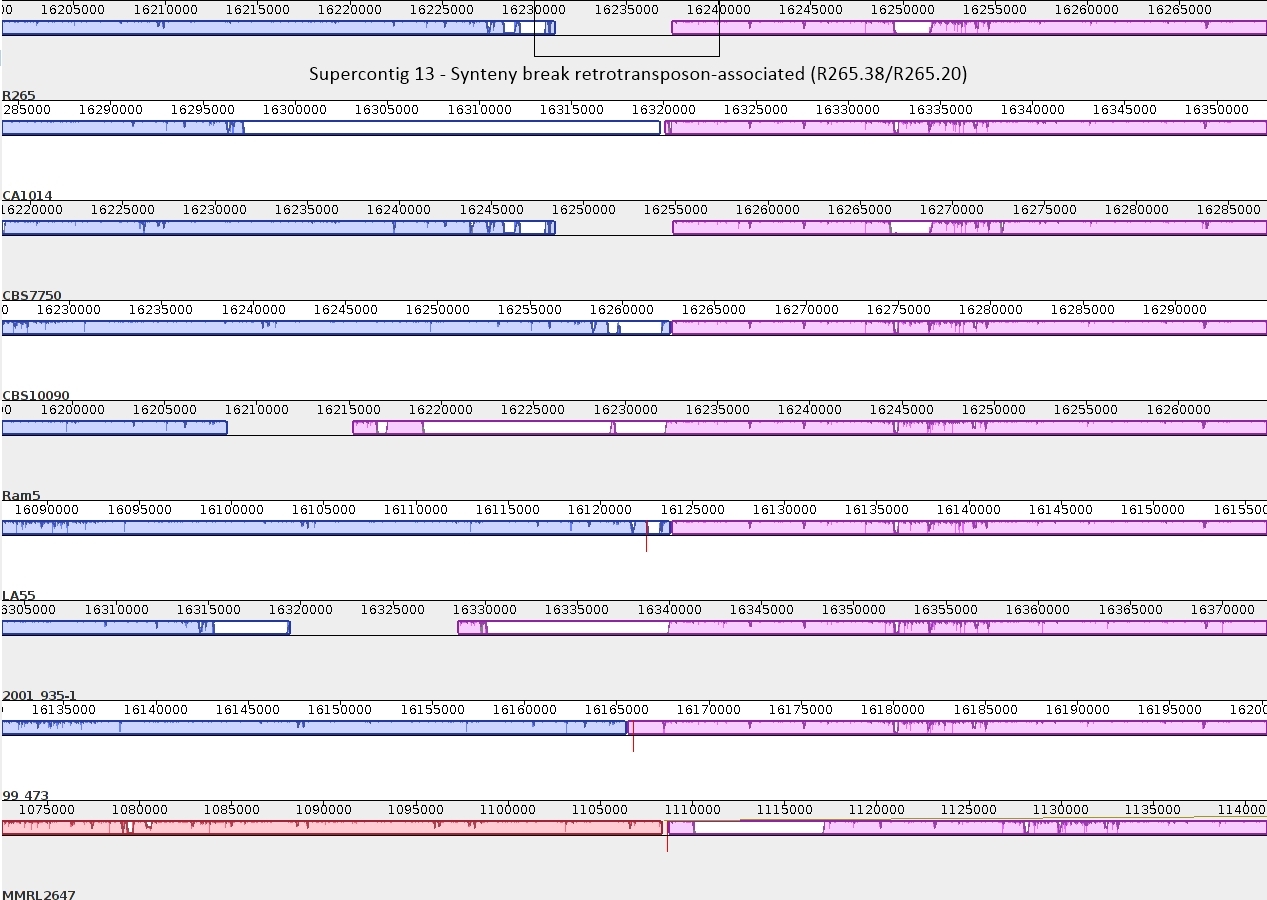

Supplement: Supplementary file 13 — Retrotransposon-associated synteny breakpoints 5 in R265. Figure shows a multiple genome alignment of VGII strains. Syntenic blocks shared by genomes are represented with the same color and are connected by lines; red lines indicate chromosome or supercontig boundaries. The location of associated retrotransposons R265.38 and R265.20 in supercontig 13 of R265 strain is represented by the black rectangle. Microsynteny disruption by retrotransposon insertion can be observed as white spaces inside blocks. (JPG 487 kb) [file 12864_2017_3688_MOESM13_ESM.jpg]

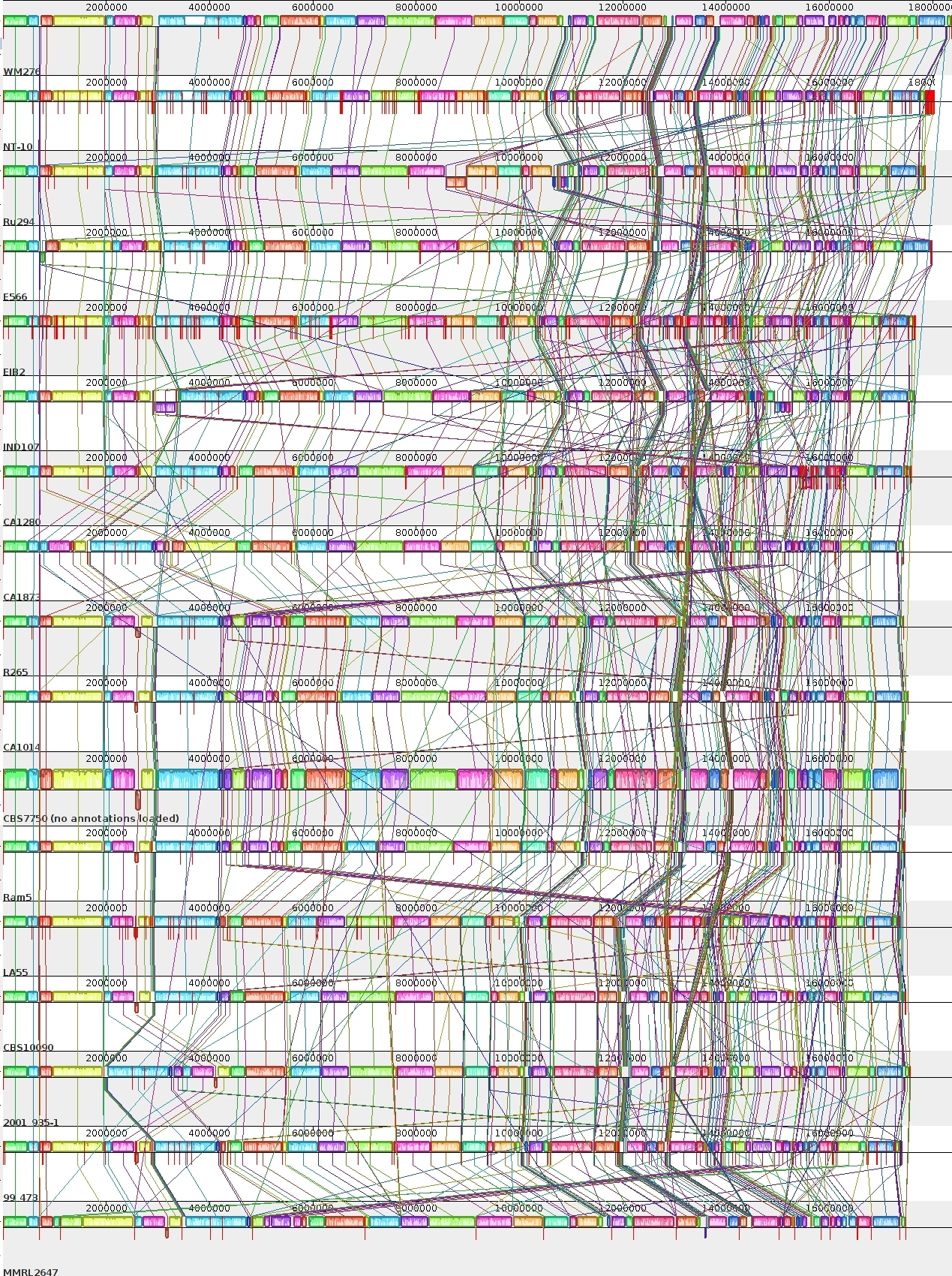

Supplement: Supplementary file 14 — VGII multiple genome alignment. Multiple genome alignment of strains from VGII molecular type with Mauve aligner. Syntenic blocks shared by genomes are represented with the same color and are connected by lines; red lines indicate chromosome or supercontig boundaries. (JPG 2507 kb) [file 12864_2017_3688_MOESM14_ESM.jpg]

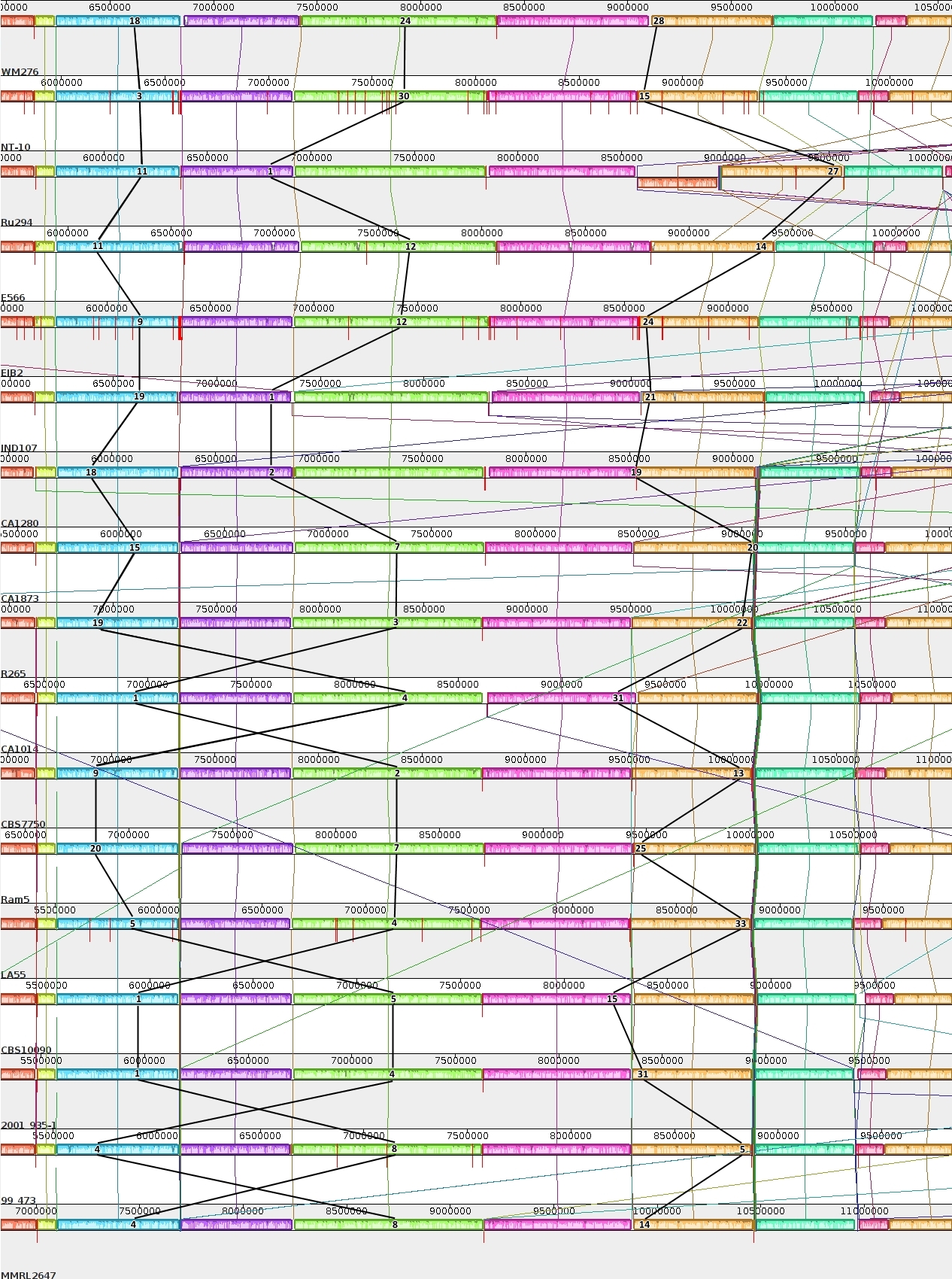

Supplement: Supplementary file 15 — Synteny analysis of R265.3, R265.19 and R265.22 orthologous sequences. Localization of orthologous retrotransposons of R265.3, R265.19, and R265.22 sequences are indicated by black lines in a multiple genome alignment with strains of VGI, VGII, VGIII, and VGIV molecular types. Orthologous sequences in each strain are named by the retrotransposon number. Syntenic blocks shared by genomes are represented with the same color and are connected by lines; red lines indicate chromosome or supercontig boundaries. (JPG 1397 kb) [file 12864_2017_3688_MOESM15_ESM.jpg]

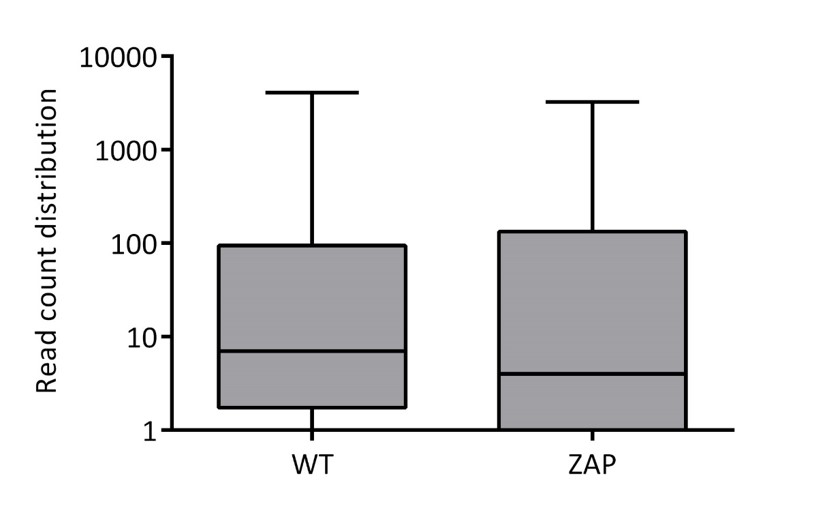

Supplement: Supplementary file 17 — Read counts values of retrotransposon sequences. Raw read counts distribution of retrotransposon sequences in WT and ZAP1 conditions. Distribution values are shown in a log scale. (JPG 27 kb) [file 12864_2017_3688_MOESM17_ESM.jpg]

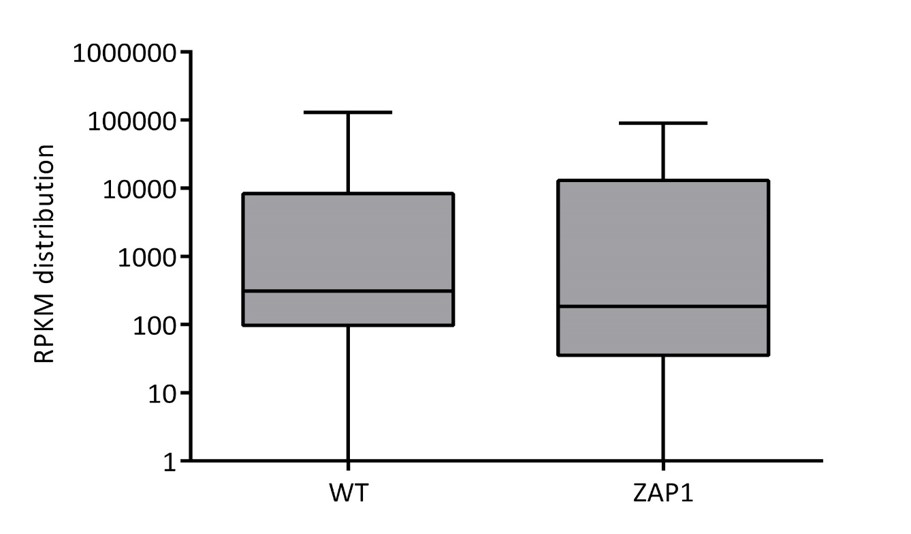

Supplement: Supplementary file 18 — RPKM values of retrotransposon sequences. RPKM distribution of retrotransposon sequences in WT and ZAP1 conditions. Distribution values are shown in a log scale. (JPG 31 kb) [file 12864_2017_3688_MOESM18_ESM.jpg]

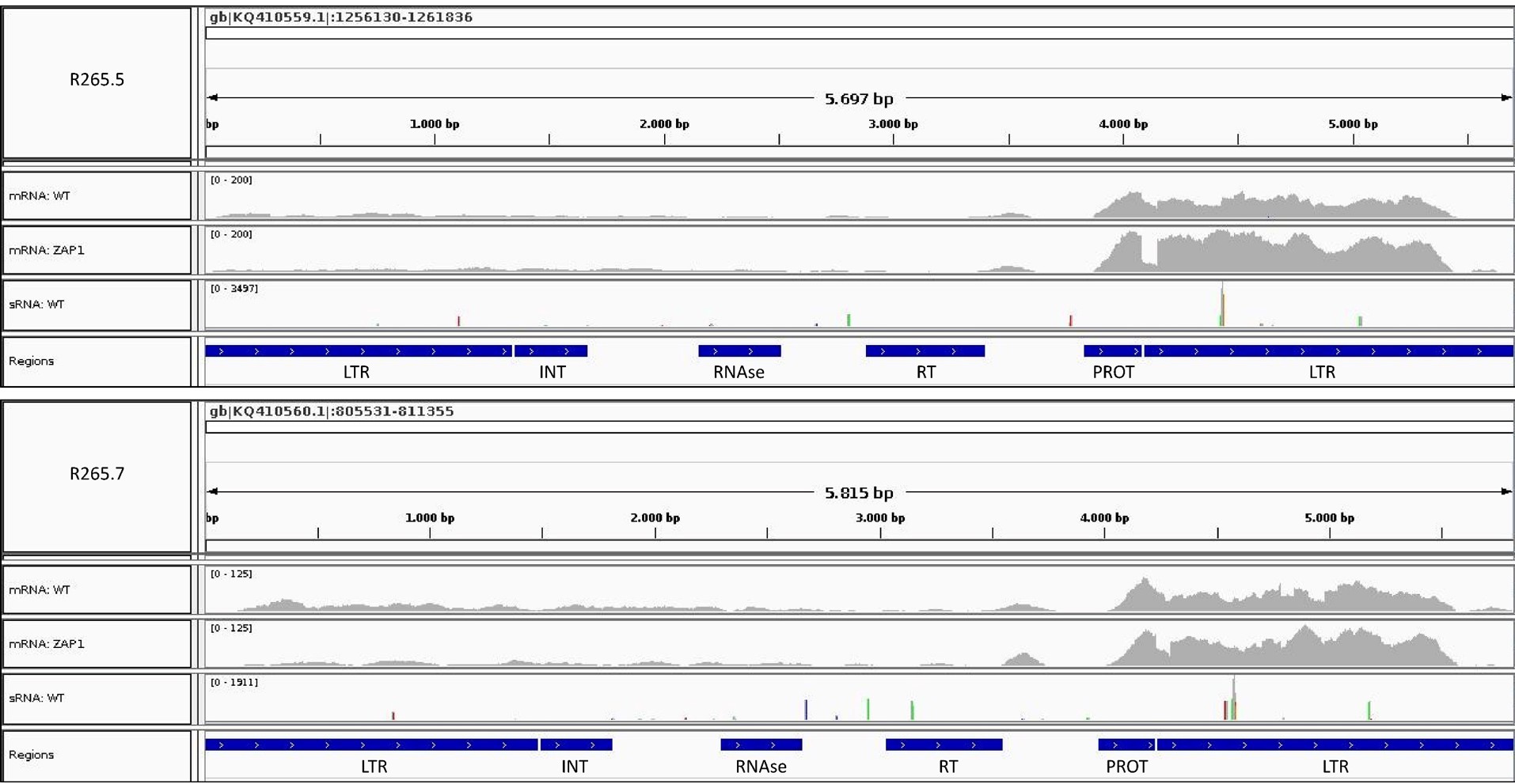

Supplement: Supplementary file 19 — Reads mapping profile of in full-length retrotransposons. Visualization of mapped reads profile from mRNA and sRNA libraries in full-length retrotransposons R265.5 and R265.7. Abbreviations: Long Terminal Repeats (LTR), Integrase (INT), Ribonuclease H (RNAse), Reverse Transcriptase (RT), Aspartyl Protease (PROT). (JPG 289 kb) [file 12864_2017_3688_MOESM19_ESM.jpg]

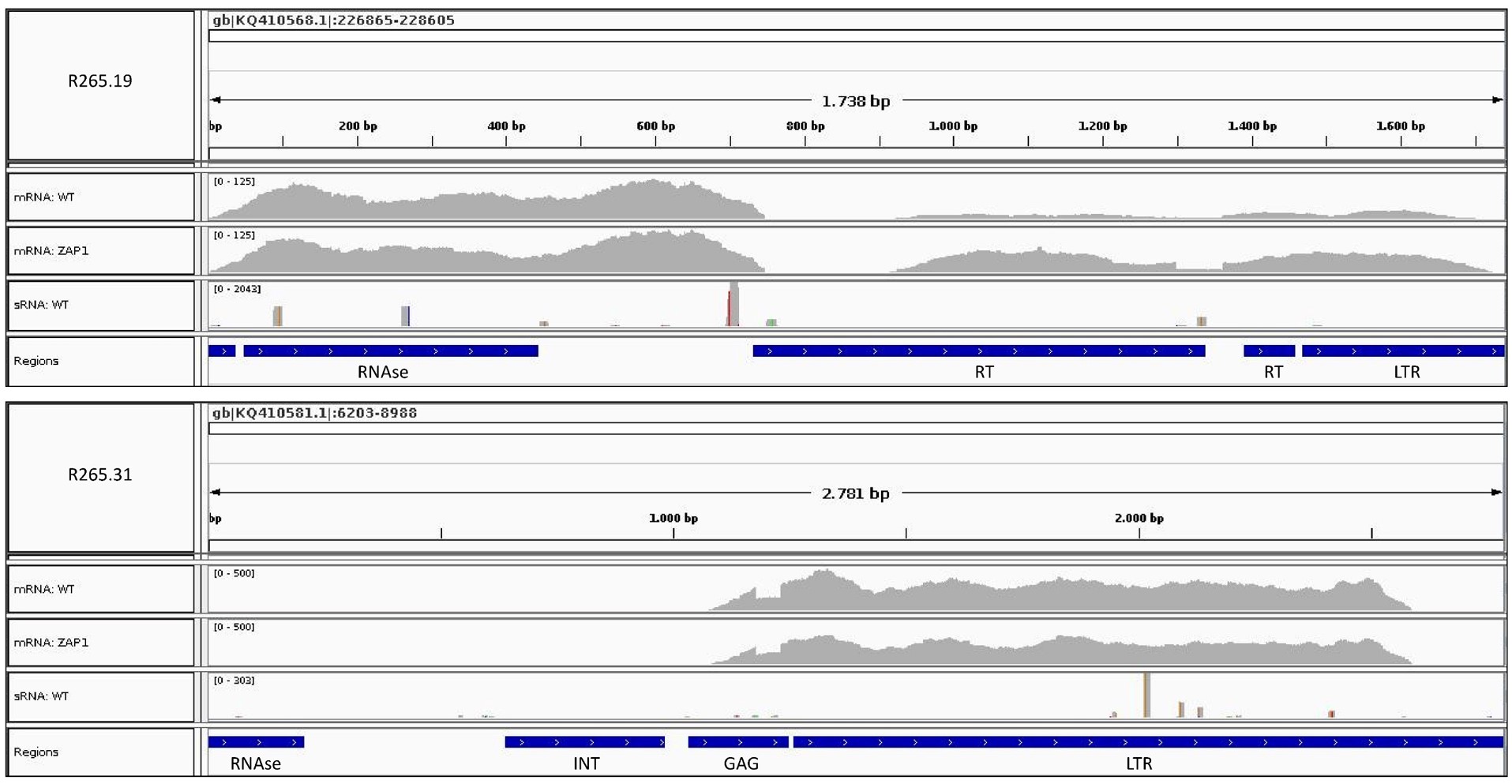

Supplement: Supplementary file 20 — Reads mapping profile of highly expressed retrotransposons. Visualization of mapped reads profile from mRNA and sRNA libraries in highly expressed sequences R265.19 and R265.31. Abbreviations: Long Terminal Repeats (LTR), Integrase (INT), Ribonuclease H (RNAse), Reverse Transcriptase (RT), Aspartyl Protease (PROT). (JPG 285 kb) [file 12864_2017_3688_MOESM20_ESM.jpg]

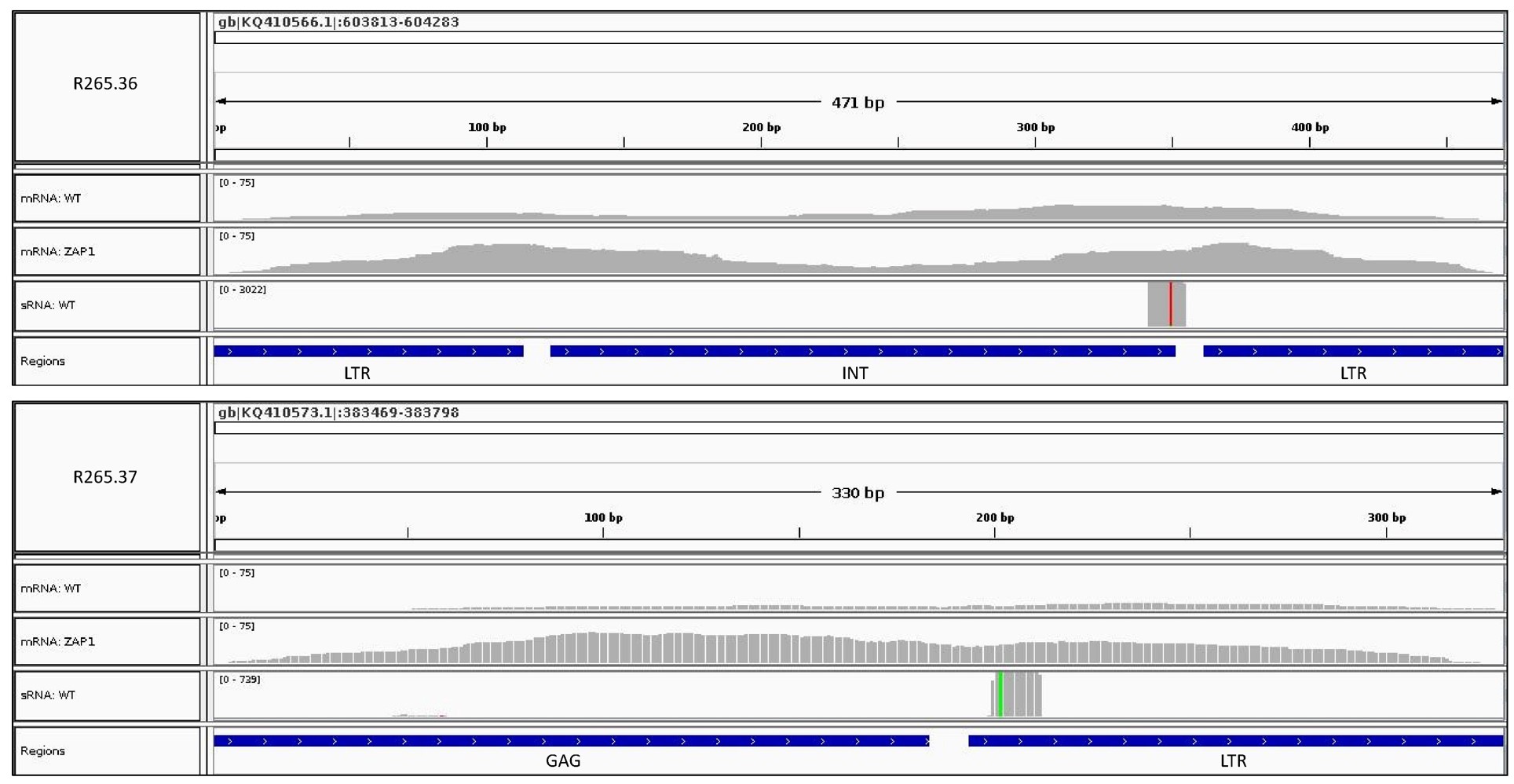

Supplement: Supplementary file 21 — Reads mapping profile of highly repressed retrotransposons. Visualization of mapped reads profile from mRNA and sRNA libraries in highly repressed sequences R265.36 and R265.37. Abbreviations: Long Terminal Repeats (LTR), Integrase (INT), Ribonuclease H (RNAse), Reverse Transcriptase (RT), Aspartyl Protease (PROT). (JPG 287 kb) [file 12864_2017_3688_MOESM21_ESM.jpg]
